# Bats as putative *Zaire ebolavirus* reservoir hosts and their habitat suitability in Africa

**DOI:** 10.1038/s41598-020-71226-0

**Published:** 2020-08-31

**Authors:** Lisa K. Koch, Sarah Cunze, Judith Kochmann, Sven Klimpel

**Affiliations:** 1 Institute for Ecology, Evolution and Diversity, Goethe-University, Max-von-Laue-Str. 13, 60438 Frankfurt/Main, Germany; 2 Senckenberg Biodiversity and Climate Research Centre, Senckenberg Gesellschaft für Naturforschung, 60438 Frankfurt/Main, Germany

**Keywords:** Ecological modelling, Ecological modelling, Ecology, Ecology, Diseases, Infectious diseases, Viral infection

## Abstract

The genus *Ebolavirus* comprises some of the deadliest viruses for primates and humans and associated disease outbreaks are increasing in Africa. Different evidence suggests that bats are putative reservoir hosts and play a major role in the transmission cycle of these filoviruses. Thus, detailed knowledge about their distribution might improve risk estimations of where future disease outbreaks might occur. A MaxEnt niche modelling approach based on climatic variables and land cover was used to investigate the potential distribution of 9 bat species associated to the *Zaire ebolavirus*. This viral species has led to major Ebola outbreaks in Africa and is known for causing high mortalities. Modelling results suggest suitable areas mainly in the areas near the coasts of West Africa with extensions into Central Africa, where almost all of the 9 species studied find suitable habitat conditions. Previous spillover events and outbreak sites of the virus are covered by the modelled distribution of 3 bat species that have been tested positive for the virus not only using serology tests but also PCR methods. Modelling the habitat suitability of the bats is an important step that can benefit public information campaigns and may ultimately help control future outbreaks of the disease.

## Introduction

Today, many infectious diseases occurring in humans are zoonotic and originate from infected wild animals^1^. Among these emerging diseases, diseases caused by filoviruses pose major health threats, since these viruses belong to the most lethal primate pathogens with average death rates of 50%^2,3^. In recent years, the genus *Ebolavirus* has gained much attention. The genus *Ebolavirus* was first identified in 1976 in the area of present-day Democratic Republic of the Congo (near the Ebola River) and the species of this genus are endemic in at least 14 African countries today.

Until today, five viral species of the genus *Ebolavirus*, namely the *Zaire ebolavirus* (ZEBOV), the *Sudan ebolavirus*, the *Taï Forest ebolavirus*, the *Bundibugyo ebolavirus* and the *Reston ebolavirus* are known. While the first four species are assumed to be endemic to Africa and have led to numerous disease outbreaks in several African countries so far^4,5^, *Reston ebolavirus* is probably native to Asian countries, e.g. the Philippines and China^3,6,7^. Similar to the Marburg virus, the different species within the genus *Ebolavirus*, except *Reston ebolavirus*, cause haemorrhagic fever, which includes various symptoms like fever, vomiting, diarrhoea and muscle pain^4,8^.

Besides their common symptoms, the five viral species differ in their pathogenicity, from asymptotic infections caused by *Reston ebolavirus* in humans to high mortality rates of up to 77–88% in case of ZEBOV infections^6,9–11^.

The largest outbreak of the Ebola virus disease (EVD) until today occurred in Western Africa between 2014 and 2016. Starting in December 2013 with a probable spillover of the virus from infected bats^12^, the epidemic spread to other parts of Africa. The largest impact and number of EVD cases were recorded in Guinea, Sierra Leone and Liberia. During this epidemic, over 15,000 laboratory-confirmed Ebola cases and over 11,000 deaths were reported^4,8^. In addition, the outbreak that has started in 2018 in the Democratic Republic of the Congo (North Kivu Province) and the surrounding countries evidently suggests that rapid surveys and further investigations are ever more relevant. Single Ebola cases have also been reported from Europe (e.g. Spain or the Netherlands) and the USA^5,8^ and can be linked to previous stays or aid missions in the affected areas.

Until today, the transmission cycle of ZEBOV, especially within the animal reservoirs, has not been fully understood. Although many of the human Ebola outbreaks in Africa can be linked to infected great apes and duikers, these animals are not regarded as the natural reservoir hosts of the virus due to their high mortality rates^6,13,14^. Since the virus’ first detection more than 40 years ago, a number of possible reservoir hosts (including bats, birds, reptiles, molluscs, arthropods and plants) have been under consideration for potential reservoir hosts^5,6,15,16^. Peterson et al. suggested that an Ebola reservoir host is likely to be a small mammal, which would not show many symptoms^17^. Additionally, some studies revealed that among mammalian taxa (with the exception of rodents), bats seem to be a major reservoir hosts bearing a large number of zoonotic pathogens, e.g. viruses like the Marburg virus or Hendra and Nipah viruses^18,19^. In particular, the long-term infection and asymptomatic survival following an infection with the virus is considered a key aspect for reservoir hosts and may indicate a co-evolution between the reservoir and the virus^13,17,19,20^. In a laboratory experiment by Swanepoel et al., the authors showed that while plants, reptiles, invertebrates and some vertebrates had been refractory to the infections in experimental inoculation, ZEBOV was able to replicate in three bat species (*Tadarida condylura* (= *Mops condylurus*), *T. pumila*, and *Epomophorus wahlbergi*)^6^. In additional laboratory experiments, the authors could also isolate the virus in the faeces of these bats 21 days after the inoculation of the virus^6^. Until today, antibodies against the different Ebola species have been detected in at least 14 bat species, including 9 species which had antibodies against ZEBOV (*Eidolon helvum*, *Epomophorus gambianus*, *Lissonycteris angolensis*, *Micropteropus pusillus*, *Mops condylurus*, *Rousettus aegyptiacus, Epomops franqueti, Hypsignathus monstrosus*, and *Myonycteris torquata*). Whereas these 9 bat species had been all tested positive for ZEBOV using serology methods, only *Epomops franqueti, Hypsignathus monstrosus* and *Myonycteris torquata* had additionally been tested positive by PCR methods^9,15,16,21–24^. PCR evidences for *Rousettus aegyptiacus* have been ambiguous and inconsistent^16,20,25^.

From those bat species that had been shown to be susceptible for ZEBOV, the virus could then infect other animals such as non-human primates, duikers (antelopes) or humans. Once the virus has spilled over from a wild animal to a human, it can be transmitted from human to human^4,5^. It has been postulated that humans might become infected with the virus through close contact with blood or other body fluids from infected persons or when handling or eating so-called bushmeat form e.g. bats roosting in or near to human dwellings^13,26–28^.

The distribution of a virus is generally restricted to the distribution of the natural reservoir host^17^. In the case of Ebola, current evidence suggests that bats might play a major role in the transmission cycle of the filoviruses and might act as reservoir hosts. The approach taken here was to model their habitat suitability and compare their potential distribution with previous spill over events. We modelled the habitat suitability for the 9 species associated with ZEBOV due to the high mortality rate associated with this viral species and its presumed responsibility for most outbreaks in Africa^4,29^.

## Results

**Figure 1:**
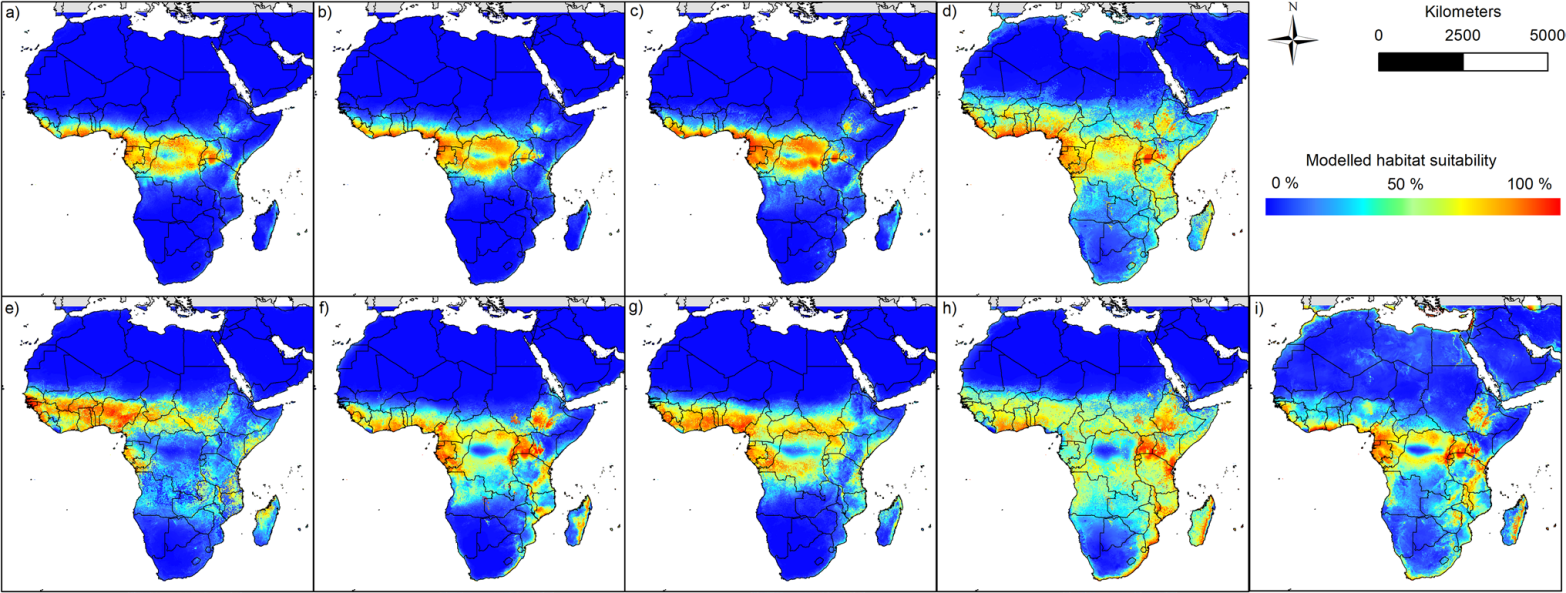
Multi-coloured modelling results of the nine bat species: (**a**) *Epomops franqueti*, (**b**) *Hypsignathus monstrosus*, (**c**) *Myonycteris torquata*, (**d**) *Eidolon helvum*, (**e**) *Epomophorus gambianus*, (**f**) *Lissonycteris angolensis*, (**g**) *Micropteropus pusillus*, (**h**) *Mops condylurus*, (**i**) *Rousettus aegyptiacus*. The maps were generated using Esri ArcGIS 10.6 (https://www.esri.com/en-us/home)^67^.

**Figure 2:**
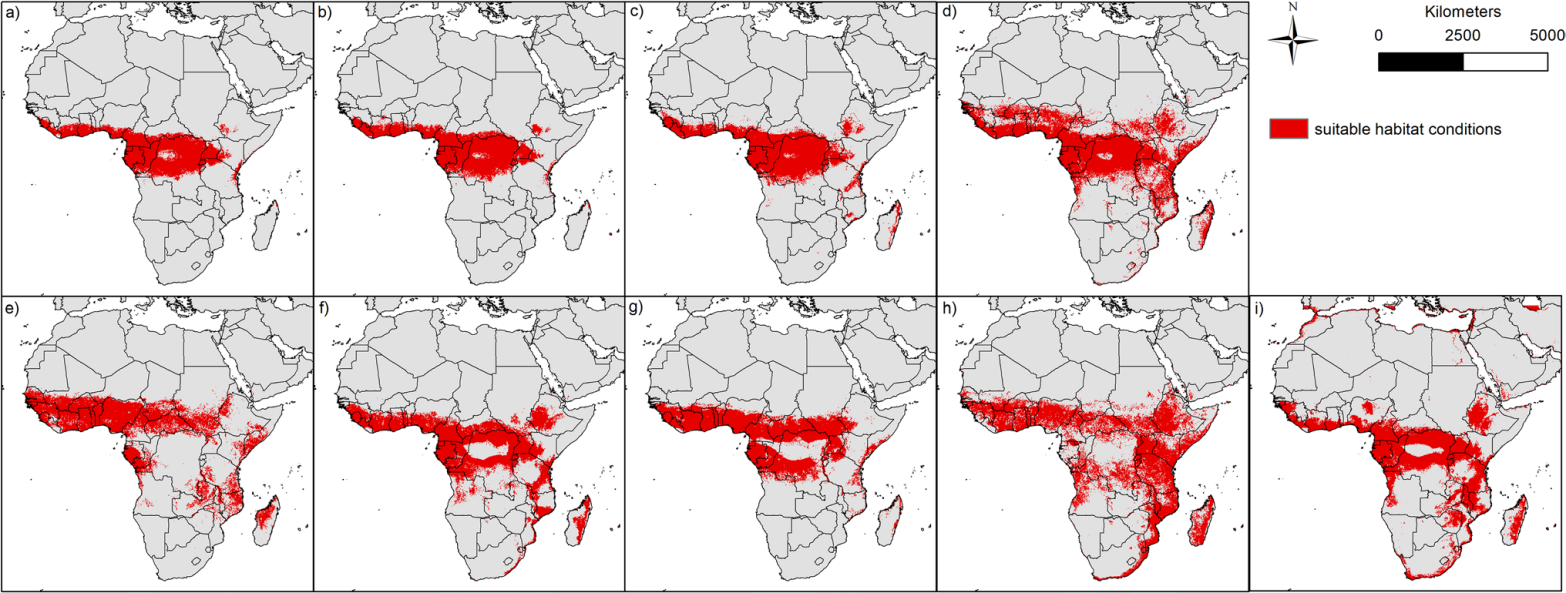
Binary modelling results of the nine bat species: (**a**) *Epomops franqueti*, (**b**) *Hypsignathus monstrosus*, (**c**) *Myonycteris torquata*, (**d**) *Eidolon helvum*, (**e**) *Epomophorus gambianus*, (**f**) *Lissonycteris angolensis*, (**g**) *Micropteropus pusillus*, (**h**) *Mops condylurus*, (**i**) *Rousettus aegyptiacus*. The maps were generated using Esri ArcGIS 10.6 (https://www.esri.com/en-us/home)^67^.

The generated habitat suitability models captured the occurrence data of the bat species to large parts, with good to very good AUC value > 0.84 (see Supplementary Material S4). Suitable habitats for the putative reservoir species currently prevail from Western to Eastern Africa, including large parts of Central Africa (see Figs. 1 and 2). More specifically, suitable areas are located below the Sahara and range from Guinea, Sierra Leone and Liberia in the west across the Central African Republic, Republic of the Congo and the Democratic Republic of the Congo, to Sudan and Uganda in the east. For some species (see *Rousettus aegyptiacus* or *Mops condylurus*) the areas even extend to the southern parts of Africa (e.g. east coast of South Africa). According to the models, no favourable habitat conditions prevail in the large deserts of Africa (e.g. Sahara or Namib), with only a very small number of occurrence data being recorded in those regions (Supplementary Materials S1 and S3). In addition, the habitat suitability of some species (e.g. *Epomophorus gambianus* and *Mops condylurus*) in the Congo Basin is significantly lower than in the surrounding area.

**Figure 3:**
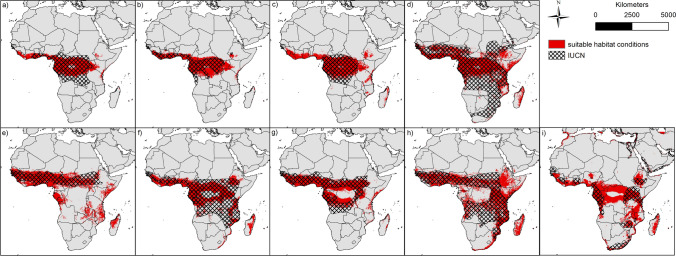
Binary modelling results of the nine bat species (red) and current status of distribution according to IUCN (hatched): (**a**) *Epomops franqueti*, (**b**) *Hypsignathus monstrosus*, (**c**) *Myonycteris torquata*, (**d**) *Eidolon helvum*, (**e**) *Epomophorus gambianus*, (**f**) *Lissonycteris angolensis*, (**g**) *Micropteropus pusillus*, (**h**) *Mops condylurus*, (**i**) *Rousettus aegyptiacus*. The maps were generated using Esri ArcGIS 10.6 (https://www.esri.com/en-us/home)^67^.

Comparing the currently assumed distribution of bat species as represented by the IUCN polygons (Fig. 3), our modelling results mostly show a match for all species. Only small areas in southern West Africa (see *Myonycteris torquata*), the Congo Basin (see *Rousettus aegyptiacus*) and Madagascar (see e.g. *Mops condylurus*) are not covered by the IUCN polygons, although they have suitable conditions according to our models.

Based on their modelled habitat suitability (Fig. 1), the different bat species can be classified into different groups. The suitable habitats of the first group (*Epomops franqueti* (Fig. 1a), *Hypsignathus monstrosus* (Fig. 1b) and *Myonycteris torquata* (Fig. 1c)) range from the areas near the coasts of West and Central Africa and from there further inland. In the east, suitable modelled areas reach up to western Kenya and Tanzania. Compared to the other two groups, the modelled potential distribution to the east and south does not extend very far. The second group, which includes the species *Epomophorus gambianus* (Fig. 1e), *Lissonycteris angolensis* (Fig. 1f), *Micropteropus pusillus* (Fig. 1g) and *Rousettus aegyptiacus* (Fig. 1i), has a broader modelled range compared to the first group. The areas modelled suitable for this group extend in the west to the southern regions of Mali and Burkina Faso. According to the modelling results, this group also finds more suitable areas in Central Africa, which partly extend to the north of Angola in the south, where *Rousettus aegyptiacus* already occurs (see Supplementary Material S1). Towards the east, the suitable habitats of this group are also rather small, and hardly reach areas in Ethiopia and Somalia. The third group, consisting of *Eidolon helvum* (Fig. 1d) and *Mops condylurus* (Fig. 1h)*,* finds suitable conditions in large parts of Africa. The areas modelled suitable for this group exceed areas of the other two groups, especially in the east and the south and range from northern areas directly south of the Sahara to the countries of Mozambique and South Africa, where both species had already been detected (Supplementary Material S1). Considering an east–west direction, the suitable habitats of the species of this group extend from Somalia and Ethiopia in the east to Guinea and Sierra Leone in the west.

**Figure 4:**
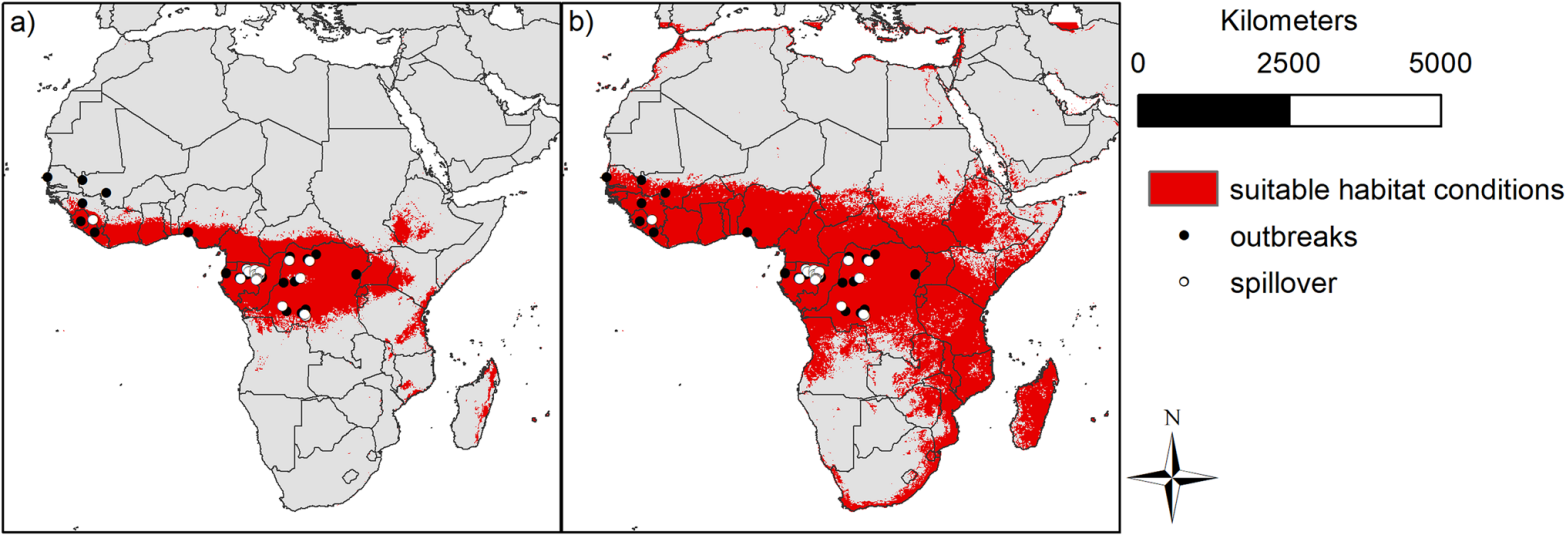
Binary modelling results of bat species (red) and previous ZEBOV spillover (white circles) and outbreak events (black circles) for the CDC and Judson et al.^65,66^: (**a**) for the 3 bat species tested positive for ZEBOV by serology and PCR method, (**b**) for all 9 investigated bat species tested serologically positive for ZEBOV. The maps were generated using Esri ArcGIS 10.6 (https://www.esri.com/en-us/home)^67^.

In addition to the modelling results of the single species, we combined all binary outputs for the 3 species that had been tested positive by serology and PCR methods (Fig. 4a) as well as for all 9 species, including the previous three species and the six species that had only been tested ZEBOV positive by serology (Fig. 4b). This was done in order to better estimate the total extent of the area with potentially suitable habitats and to test whether the extent would cover areas where previous spillover events and outbreak sites of the ZEBOV were recorded (Fig. 4a,b). The spillover events and outbreak sites considered here are located in Central Africa, especially in Gabon, the Republic of the Congo and the Democratic Republic of the Congo and Western Africa (e.g. Liberia and Sierra Leone). The modelled habitat suitability of the subset of the 3 species nearly covers all considered previous spillover event and outbreak sites. Considering all 9 putative reservoir species the area modelled as suitable is even larger. The area extends from Senegal, Gambia and Liberia in West Africa, across the southern parts of Mali, Niger and Chad to the eastern parts of Ethiopia and Somalia. In the south, the suitable area for the 9 species extends to the northern parts of Angola and Zambia, along the east coast even to South Africa.

Regarding the land cover, all of the investigated putative reservoir species find suitable habitats in the areas of the tropical rainforest near the coasts of West and Central Africa and further inland in Central Africa. In the south, the area extends into the southern woodlands and savannah areas for some species like *Mops condylurus* or *Rousettus aegyptiacus*. For the majority of species, the first two variables explaining more than 70–80% of the variance, are mostly land cover and temperature seasonality (Bio04). The land cover variable alone is often responsible for more than 60% of the explained variance (Supplementary Material S4) and the species show different patterns with regard to the land cover types modelled to be suitable (Supplementary Material S5). Other variables such as the annual mean temperature (Bio01) and precipitation in the wettest month (Bio13) have very little explanatory value for the variance in the model and values range between 0 and 3.94% and between 0.1 and 7.25% for Bio1 and Bio13, respectively.

## Discussion

In recent years, bats have increasingly been recognized as putative reservoir hosts for several viruses including different species within the genus *Ebolavirus* and due to the associated health threat they became the focus of many studies. Since the species of the genus *Ebolavirus* are highly dependent on the reservoir host, knowledge about their reservoir host’s distribution is important. The aim of the study was to investigate the habitat suitability for 9 currently known putative reservoir species of the deadly ZEBOV in Africa, with 3 of these species having been tested positive for ZEBOV not only by serology but also PCR methods.

All the models in this study estimated the distribution of the investigated species well, with suitable habitat conditions in the sub-Saharan regions of West and Central Africa, especially in the areas of forests and woodlands. For some species, suitable habitats even extend to the southeast of Africa and the eastern coast (e.g. *Eidolon helvum* and *Mops condylurus*), which is in accordance with their currently known distribution (see Supplementary Material S1). In the Congo Basin, the models of many species assume poor habitat conditions. These poor habitat conditions are paralleled by the comparatively few occurrence records found in this area (Supplementary Materials S1 and S3)^30,31^. This might be explained by unique habitat conditions, particularly in the equatorial regions in the centre of the Congo basin. The Congo Basin is mainly covered with forest and with about 1.7 million km^2^ it is the second largest rainforest in the world. In general, the Congo Basin is to be classified as an intermediate tropical region, with average rainfall between 1,000 and 1,750 mm per year, however, equatorial areas differ from those amounts^32^. In particular, the heavy rainfall in the equatorial areas with an average of 2000 mm and the lower temperatures compared to the ambient areas could pose a challenge for the putative reservoir hosts. On the one hand, it is assumed that heavy rainfall could lead to energetic costs for bats. On the other hand, flight characteristics of insects are influenced by frequent and heavy rainfall, which could indirectly affect the habitat suitability of the insectivorous species like (*Mops condylurus*) in these regions^30,33,34^. Although the precipitation variables in the model of *Mops condylurus* are comparatively important explanatory variables, they do not have a very high explanatory power in general compared to the other species. Erickson and West (2002) could detect a strong negative correlation between the flight activity of insectivorous bats and low temperatures^35^. Thus, the comparatively lower and less variable temperatures in the equatorial areas of the basin (~ 23 to 24 °C mean annual temperature) might also influence the habitat suitability in this area^32^. In the case of *Mops condylurus*, it is known that this species prefers temperatures of about 35–40 °C in its roosting areas, similar to their basal metabolic rate^30^. Similar results were found for the frugivorous species *Rousettus aegyptiacus*. This species was found in resting areas with temperatures between 29–35 °C, which also corresponds to its metabolic optimum^30^. However, the poor habitat suitability in this region might also be due to a sampling bias or a general lack of scientific occurrence data from the equatorial rainforest^36^, occurrences from this the region might have been underestimated in the model.

The modelled habitat suitabilities are also in good accordance with the currently assumed distribution of the nine bat species according to the IUCN. Differences between the IUCN polygons and the model (e.g. in Madagascar) may indicate dispersal barriers^36^. For example, although the habitats in Madagascar are modelled suitable for the species, they are very likely unable to reach them. Other discrepancies between the IUCN maps and our habitat suitability maps might indicate that species occupy a larger range but have not been recorded in parts of this range. The available distribution data might therefore underestimate the actual distribution. In this case, our maps would provide a better estimate of the actual distribution of the putative Ebola reservoir hosts, and help identify regions where Ebola might occur. However, the observed differences could also arise from the fact that our models identify areas where habitats are suitable, but where the species are not present. The absence of species in these areas despite modelled habitat suitability could be due to the dispersal limitations or lacking accessibility of the sites (e.g. Madagascar), or due to the fact that relevant factors that exclude the occurrence of a species were not considered in the model, e.g. availability of certain food types or interspecific competition with other bat species.

The areas modelled as suitable in our study are also in accordance with results of former studies^29,34^. In comparison to these studies, we used a more recent data set of occurrences (until 2019) and climate variables (worldclim data containing the average for the years 1970–2000)^37^. Moreover, this study is not only based on modelling using climatic factors, but also uses land cover variables, which might help to better assess the bats' habitat requirements.

Land cover plays an important role in almost all models (variable contribution with mostly over 60%) and therefore has an important explanatory effect. This might be because the bat species require trees as resting and roosting places, but also for feeding, since eight of the nine investigated species mainly feed on fruits^30,34^. More specifically, the results indicate that bats mainly find suitable conditions in the forests (e.g. land cover classes 40 and 170 in Supplementary Material S5). However, all of the investigated species also show a strong association with artificial surfaces and associated areas (land cover class 190). For many of the investigated bat species this could be explained by the fact that these species live or roost close to urban areas and human dwellings (e.g. *Eidolon helvum* or *Rousettus aegyptiacus*)^30,38^.

Bat species roosting in great densities in crowded colonies might facilitate intra- and interspecies transmission of viral infections and increase the infection risk in the surrounding areas^30,38–41^. Generally, there is a high risk of accumulation of viruses and parasites in large groups of bats and individuals might be more vulnerable to infections due to their close proximity^26,40–43^. One species usually roosting in large groups (sometimes up to more than one million bats) is *Eidolon helvum*^30,38^. This species is one of the most hunted species and considered a main source of bushmeat for inhabitants of West and Central Africa, which makes it an important species for a virus spillover from bats to human populations^30,38,44^. Although the causes of EVD outbreaks and the associated factors are still largely unknown, an epidemiological study was able to show a link between an outbreak of EVD in the Democratic Republic of the Congo and an increased flight activity and aggregation of bats during migration in combination with subsequent hunting of these bats^20,45^. *Eidolon helvum* and *Mops condylurus* are migratory species and their activity ranges vary between 1,190 and 1,437 ha. They also have large foraging distances of up to 4.8 km, and *Eidolon helvum* sometimes migrates over distances between 250 and 2000 km per season^30,38,41^. Migratory bat species might therefore promote and facilitate the viral spread/transmission of ZEBOV, even across biomes or continents^41^. Given that the migratory routes and intrinsic drivers of the bats are largely unknown, future behavioural and ecological studies are necessary and could also benefit a better understanding of how to limit a possible spread of the virus. In order to examine the migration behaviour more effectively, modelling results with higher temporal resolution may also be useful. Such results might highlight suitable habitats of the species over the timespan of a year and thus, facilitate sampling on the one hand and help draw possible conclusions on migration behaviour on the other hand. These facts might indicate an important role of *E. helvum* in the transmission of the virus, however Ng et al. could experimentally show that this species has not been much susceptible to the virus^46^. Their experiment might therefore indicate that *E. helvum* is genetically not well suited for cellular virus infections and the antibodies previously detected in this species could have been generated against a related filovirus or, since their experiments have been based on in-vitro cell culture data, they might not necessarily reflect the situation in nature.

The modelled distribution of the three species that had been tested positive for ZEBOV by different methods (serology and PCR) overlaps with nearly all of the previous spillover events and outbreak sites extracted from literature (Fig. 4a) located in central African, seemingly supporting the importance of *Epomops franqueti, Hypsignathus monstrosus* and *Myonycteris torquata*, as putative ZEBOV reservoirs. This assumption is also in accordance with the results of a study by Pourrut et al., who were able to detect ZEBOV-specific antibodies serologically in all three species (namely *Epomops franqueti*, *Hypsignathus monstrosus* and *Myonycteris torquata*) during the outbreaks in 2001 and 2003 in Gabon and in the Republic of the Congo^16^. Whether the other six other species (*Eidolon helvum*, *Epomophorus gambianus*, *Lissonycteris angolensis*, *Micropteropus pusillus*, *Mops condylurus* and *Rousettus aegyptiacus*), which had only been tested positive for ZEBOV using serology tests but are partly overlapping in their distribution with the other 3 bat species, might similarly act a putative reservoir hosts, requires further molecular investigations. However, since cross-reactivity of antibodies cannot be excluded, the detection of ZEBOV-specific antibodies could also indicate a related but perhaps not yet discovered virus strain, rather than actual antibodies against the *Zaire ebolavirus*^47,48^.

In addition to the putative African reservoir hosts, it could also be beneficial to examine other bat species outside Africa regarding their potential to act as reservoir hosts for the *Zaire ebolavirus* or other filovirus species. Until now, only a small amount (~ 15%) of the global bat species (over 1,200 species) has been examined for viruses^20,49^. One virus that should certainly be further analysed is the Lloviu virus. This filovirus was recently found in the bat species *Miniopterus schreibersii* in southern Europe^2^. However, in comparison to the Ebola species from Africa and the Philippines, which can remain asymptomatic in living bats, the Lloviu virus was only found in dead and not in living bats^2,20^.

To our knowledge, our study is the first study that investigated all currently known putative reservoir species of the deadly ZEBOV in Africa on a single species level. The models generated here reveal information about the environmental niche and the potential distribution of the bats. In relation to future outbreaks of the EVD, this information might potentially allow a better estimation of where precautionary steps and preventive actions might become necessary in the future to protect local human populations.

## Material and methods

### Occurrence data and environmental variables

In this study, we included a bat species as putative reservoir host if it had at least been serologically tested positive for ZEBOV. According to this criterion, 9 putative reservoir host species were identified. Among those, 3 had additionally been tested positive for ZEBOV by PCR method, which might further indicate their relevance as Ebola reservoir hosts^9,15,16,21–24^.

In order to not only map a broad area including all 9 putative reservoir hosts, but also a more conservative and narrow map, the three species were treated separately in an additional analysis. The occurrences of the 9 reservoir species (associated to the ZEBOV) included a total number of 3,052 data points (see Table 1).

**Table 1:** Number of occurrence records of the bat species in Africa.

Species	Number of occurrence records
*Epomops franqueti*	341
*Hypsignathus monstrosus*	219
*Myonycteris torquata*	143
*Eidolon helvum*	635
*Epomophorus gambianus*	346
*Lissonycteris angolensis*	130
*Micropteropus pusillus*	397
*Mops condylurus*	358
*Rousettus aegyptiacus*	483
Total	3,052

The corresponding references to the GBIF^50^ and African Chiroptera Reports 2018 and 2019^30,31^ occurrence data can be found in the Supplementary Material (see Supplementary Materials S1–S3).

The occurrence data within the study area (Africa and Arabian Peninsula: 37.81 to − 35.85 N and − 29.17 to 61.33 E) originate from the Global Biodiversity Information Facility (GBIF) and the African Chiroptera Reports^30,31,50^. The data was compiled in R (version 3.43, R Core Team 2017) with the R-Package “rgbif”^51,52^ and the corresponding references to the GBIF and African Chiroptera Reports occurrence data can be found in the Supplementary Material (see Supplementary Materials S2 and S3). Subsequently, the occurrence records were matched to the raster of the environmental variables with a grid size of ~ 10 km^2^.

To model the distribution of the bat species, both climate and land cover variables served as predictor variables. The climatic variables were provided by worldclim (version 2.0)^37^ and consist of interpolated empirical data, which is averaged for the years 1970–2000 with a spatial resolution of 5 arc minutes. This spatial resolution is in accordance with the resolution of about 10 km^2^ at the equator. Bat activity patterns are known to be affected by temperature and precipitation^35,53,54^. A careful pre-selection resulted in a subset of eight out of the nineteen available variables, which were considered ecologically important for the species and showed only little intercorrelation. We modelled the ecological niche of the 9 bat species using the annual mean temperature (Bio1), temperature seasonality (Bio04), max temperature of the warmest month (Bio05), min temperature of the coldest month (Bio06), the annual precipitation (Bio12), precipitation of wettest month (Bio13), precipitation of driest month (Bio14) and precipitation seasonality (Bio15). These variables reflect the mean, minima and maxima and variability of the annual temperature and precipitation patterns.

The land cover variable was extracted from the GlobCover 2009 land cover map covering Africa and the Arabian Peninsula and was provided by the European Space Agency (ESA) and the Université Catholique de Louvain^55^. This map offers a regionally-tuned MERIS (Medium Resolution Imaging Spectrometer classification) classification for the year 2009 and comprises 22 different land cover classes according to the United Nations Land Cover Classification System^55,56^. The resolution of the land cover data was set to 5 arc min in order to match the worldclim climate data and the occurrence data.

### Niche modelling and diversity maps

The habitat suitability for the 9 bat species was modelled using a basic species distribution modelling approach (also called ecological niche modelling) that combines the observed occurrences of the species with the prevailing climatic conditions and land cover. We used a maximum entropy approach conducted in the freeware MaxEnt^57^, which is one of the most commonly applied algorithms determining species distributions. This algorithm is based on presence-only data, which benefits modelling small and mobile species as their absence is difficult to determine. Moreover, the algorithm scores well in studies comparing different algorithms^58^. According to the MaxEnt default settings we only used linear, quadratic and product features and enhanced the number of iterations to 500 000 in order to ensure convergence^59^.

Multi-coloured climatic suitability was converted into binary modelling results (only suitable habitat: 1 or unsuitable habitat: 0) applying an equal training sensitivity and specificity threshold^60,61^. The threshold minimizes the difference between sensitivity and specificity as optimization criterion^62,63^. In order to compare our modelled range of the investigated bat species with their currently assumed distribution defined by the IUCN (see Supplementary Material S6), we added the IUCN polygons over our binary modelling results^64^.

To assess the potential modelled distribution of the bat species in relation to previous ZEBOV spillover and outbreak events, we used the spillover events from Judson et al. as well as the outbreak sites from the CDC website ^65,66^ and compared them with the modelled bat habitats to indicate areas where at least one species is modelled to find suitable conditions. This comparison was done for the 3 species tested positive for ZEBOV by multiple methods (Fig. 4a) and taking all nine species into account (Fig. 4b).

### Software

We used a maximum entropy approach conducted in the freeware MaxEnt^57^ to model the habitat suitability for the bat species. Further analyses were done using the statistical programme R (version 3.43)^52^ using the R-Package “rgbif”^51^ to access GBIF data ESRI ArcGIS (Release 10.6)^67^. ESRI ArcGIS was also used to generate maps.

## Supplementary information

Supplementary information.
