# Supplementary material for: Bats as putative Zaire ebolavirus reservoir hosts and their habitat suitability in Africa
Source: Sci Rep. 2020 Aug 31;10:14268. doi: 10.1038/s41598-020-71226-0 (PMC7459104; doi:10.1038/s41598-020-71226-0)
Supplement: Supplementary file 1 — Supplementary information. [file 41598_2020_71226_MOESM1_ESM.docx]

**Supplementary Materials**

**Bats as putative *Zaire ebolavirus* reservoir hosts and their habitat suitability in Africa**

Lisa K. Koch^1*^, Sarah Cunze^1^, Judith Kochmann^2^ and Sven Klimpel^1,2^

^1^ Goethe-University, Institute for Ecology, Evolution and Diversity; D60438 Frankfurt/Main, Germany

^2^ Senckenberg Gesellschaft für Naturforschung; Senckenberg Biodiversity and Climate Research Centre, D60438 Frankfurt/Main, Germany

*Corresponding author.

Goethe-University, Institute for Ecology, Evolution and Diversity; Max-von-Laue-Str. 13, D60438 Frankfurt/Main, Germany

E-Mail: l.koch@bio.uni-frankfurt.de

ORCID: https://orcid.org/0000-0002-3064-8401

**Supplementary material S1: Occurrence records from GBIF and the ACR 2018 and 2019**^1–3^.
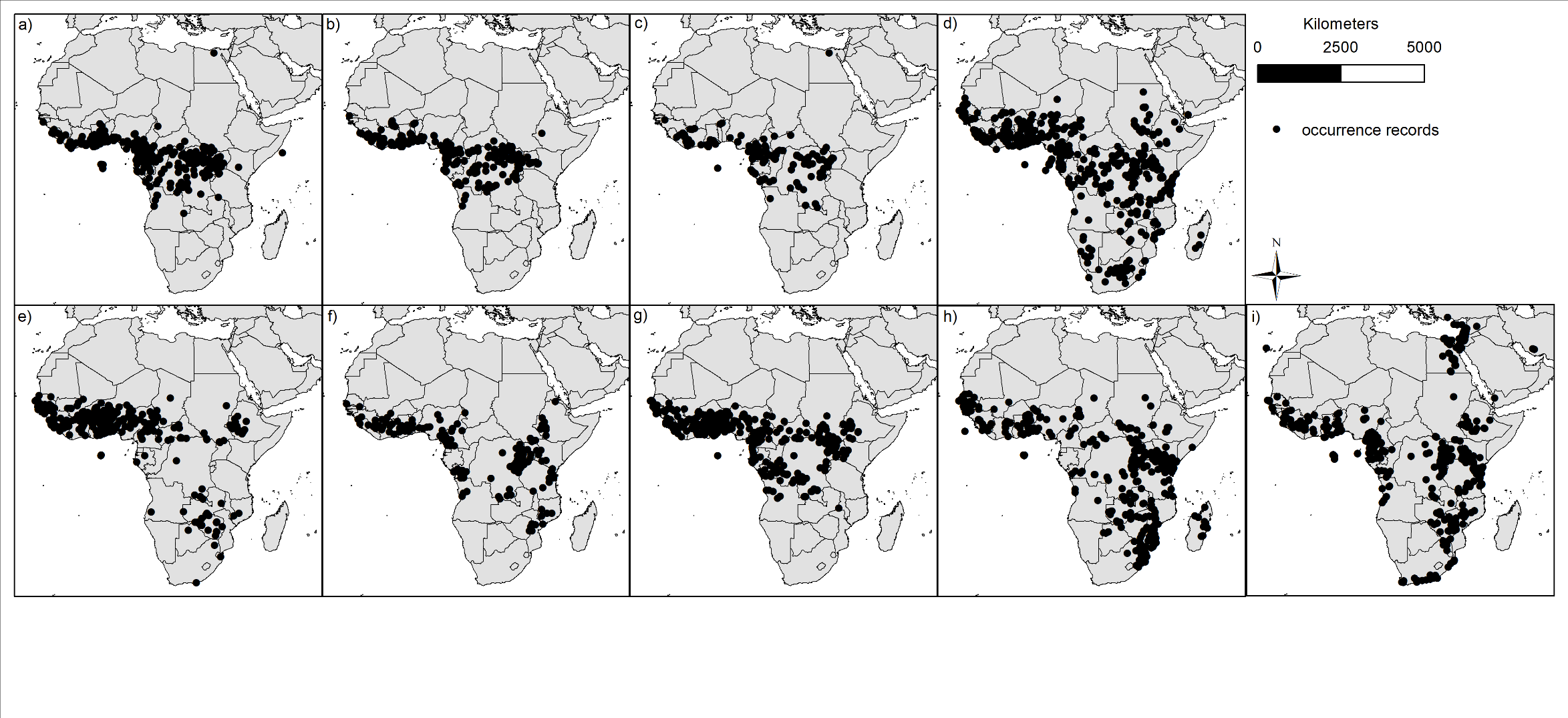


**Figure S1: Overview of the occurrence points used for modelling of the nine bat species:** a) *Epomops franqueti*, b) *Hypsignathus monstrosus*, c) *Myonycteris torquata*, d) *Eidolon helvum*, e) *Epomophorus gambianus*, f) *Lissonycteris angolensis*, g) *Micropteropus pusillus*, h) *Mops condylurus*, i) *Rousettus aegyptiacus*. The corresponding references to the GBIF and African Chiroptera Report occurrence data can be found in the supplementary material (see S2 and S3). The maps were generated using Esri ArcGIS 10.6 (https://www.esri.com/en-us/home)^4^.

**Supplementary material S2: References of the exact occurrence records from GBIF**^1^.

1. ***Eidolon helvum***

[[1]]

<<rgbif citation>>

Citation: iNaturalist.org (2018). iNaturalist Research-grade Observations.

Occurrence dataset https://doi.org/10.15468/ab3s5x accessed via

GBIF.org on 2018-08-29.. Accessed from R via rgbif

(https://github.com/ropensci/rgbif) on 2018-08-29

Rights:

[[2]]

<<rgbif citation>>

Citation: naturgucker.de. naturgucker. Occurrence dataset

https://doi.org/10.15468/uc1apo accessed via GBIF.org on 2018-08-29..

Accessed from R via rgbif (https://github.com/ropensci/rgbif) on

2018-08-29

Rights:

[[3]]

<<rgbif citation>>

Citation: Yêhouénou Tessi D R (2017). List of animalia, fungi and plant

species recorded through naturalist observations and research

activities in Benin. Data mobilized in the framework of a JRS

Biodiversity Foundation project of Benin. Laboratory of Forest Sciences

(University of Abomey-Calavi). Occurrence dataset

https://doi.org/10.15468/22kwre accessed via GBIF.org on 2018-08-29..

Accessed from R via rgbif (https://github.com/ropensci/rgbif) on

2018-08-29

Rights:

[[4]]

<<rgbif citation>>

Citation: Musila S, Syingi R, Zuhura A, Gichuki N (2018). Distribution and

abundance of different bat species in the interior of Arabuko-Sokoke

Forest and adjacent farmlands in Gede, Kilifi County, Kenya. Version

1.2. National Museums of Kenya. Occurrence dataset

https://doi.org/10.15468/zekkpx accessed via GBIF.org on 2018-08-29..

Accessed from R via rgbif (https://github.com/ropensci/rgbif) on

2018-08-29

Rights:

[[5]]

<<rgbif citation>>

Citation: Esselstyn J (2017). LSUMZ Mammals Collection. Louisiana State

University Museum of Natural Science. Occurrence dataset

https://doi.org/10.15468/wxiqf6 accessed via GBIF.org on 2018-08-29..

Accessed from R via rgbif (https://github.com/ropensci/rgbif) on

2018-08-29

Rights:

[[6]]

<<rgbif citation>>

Citation: Grant S, Ferguson A (2018). Field Museum of Natural History

(Zoology) Mammal Collection. Version 9.5. Field Museum. Occurrence

dataset https://doi.org/10.15468/n4zgxw accessed via GBIF.org on

2018-08-29.. Accessed from R via rgbif

(https://github.com/ropensci/rgbif) on 2018-08-29

Rights:

[[7]]

<<rgbif citation>>

Citation: KINGBO A, KIKI P P B (2016). Census of the animals of Benin.

Version 1.2. GBIF Benin. Occurrence dataset

https://doi.org/10.15468/h7rqo9 accessed via GBIF.org on 2018-08-29..

Accessed from R via rgbif (https://github.com/ropensci/rgbif) on

2018-08-29

Rights: Public Domain (CC0 1.0)

[[8]]

<<rgbif citation>>

Citation: MNHN - Museum national d'Histoire naturelle (2018). The mammals

collection (ZM) of the Muséum national d'Histoire naturelle (MNHN -

Paris). Version 43.77. Occurrence dataset

https://doi.org/10.15468/j0xw9i accessed via GBIF.org on 2018-08-29..

Accessed from R via rgbif (https://github.com/ropensci/rgbif) on

2018-08-29

Rights:

[[9]]

<<rgbif citation>>

Citation: European Molecular Biology Laboratory (EMBL) (2014).

Geographically tagged INSDC sequences. Occurrence dataset

https://doi.org/10.15468/cndomv accessed via GBIF.org on 2018-08-29..

Accessed from R via rgbif (https://github.com/ropensci/rgbif) on

2018-08-29

Rights:

[[10]]

<<rgbif citation>>

Citation: Flannery M, Fong J (2018). CAS Mammalogy (MAM). Version 133.136.

California Academy of Sciences. Occurrence dataset

https://doi.org/10.15468/dhbozg accessed via GBIF.org on 2018-08-29..

Accessed from R via rgbif (https://github.com/ropensci/rgbif) on

2018-08-29

Rights:

[[11]]

<<rgbif citation>>

Citation: Conroy C (2018). MVZ Mammal Collection (Arctos). Version 35.17.

Museum of Vertebrate Zoology. Occurrence dataset

https://doi.org/10.15468/uwudf9 accessed via GBIF.org on 2018-08-29..

Accessed from R via rgbif (https://github.com/ropensci/rgbif) on

2018-08-29

Rights:

[[12]]

<<rgbif citation>>

Citation: Biological Station of Doñana (CSIC) (2018). Estacion Biologica

Donana - CSIC, Mammal Collection. Occurrence dataset

https://doi.org/10.15468/fwrsxr accessed via GBIF.org on 2018-08-29..

Accessed from R via rgbif (https://github.com/ropensci/rgbif) on

2018-08-29

Rights:

[[13]]

<<rgbif citation>>

Citation: Abraczinskas L (2016). MSU Mammalogy, Ornithology and Vertebrate

Paleontology Collections. Version 8.1. Michigan State University

Museum. Occurrence dataset https://doi.org/10.15468/xypr72 accessed via

GBIF.org on 2018-08-29.. Accessed from R via rgbif

(https://github.com/ropensci/rgbif) on 2018-08-29

Rights:

[[14]]

<<rgbif citation>>

Citation: Orrell T, Hollowell T (2018). NMNH Extant Specimen Records.

Version 1.19. National Museum of Natural History, Smithsonian

Institution. Occurrence dataset https://doi.org/10.15468/hnhrg3

accessed via GBIF.org on 2018-08-29.. Accessed from R via rgbif

(https://github.com/ropensci/rgbif) on 2018-08-29

Rights:

[[15]]

<<rgbif citation>>

Citation: Millen B, Lim B (2018). Mammalogy Collection - Royal Ontario

Museum. Version 11.5. Royal Ontario Museum. Occurrence dataset

https://doi.org/10.15468/2rlrvh accessed via GBIF.org on 2018-08-29..

Accessed from R via rgbif (https://github.com/ropensci/rgbif) on

2018-08-29

Rights:

[[16]]

<<rgbif citation>>

Citation: Garner H (2016). TTU Mammals Collection. Version 9.1. Museum of

Texas Tech University (TTU). Occurrence dataset

https://doi.org/10.15468/yocqyp accessed via GBIF.org on 2018-08-29..

Accessed from R via rgbif (https://github.com/ropensci/rgbif) on

2018-08-29

Rights:

[[17]]

<<rgbif citation>>

Citation: Mateke C, Mulwanda S, Nachinga D (2018). Livingstone Museum Bat

Collection. Livingstone Museum. Occurrence dataset

https://doi.org/10.15468/i6cor7 accessed via GBIF.org on 2018-08-29..

Accessed from R via rgbif (https://github.com/ropensci/rgbif) on

2018-08-29

Rights:

[[18]]

<<rgbif citation>>

Citation: Trombone T (2016). AMNH Mammal Collections. American Museum of

Natural History. Occurrence dataset https://doi.org/10.15468/wu3poe

accessed via GBIF.org on 2018-08-29.. Accessed from R via rgbif

(https://github.com/ropensci/rgbif) on 2018-08-29

Rights:

[[19]]

<<rgbif citation>>

Citation: Bucci M (2016). UAZ Mammals. Version 5.1. University of Arizona

Museum of Natural History. Occurrence dataset

https://doi.org/10.15468/2swesj accessed via GBIF.org on 2018-08-29..

Accessed from R via rgbif (https://github.com/ropensci/rgbif) on

2018-08-29

Rights:

[[20]]

<<rgbif citation>>

Citation: Muséum d'histoire naturelle de la Ville de Genève - MHNG. Mammals

housed at MHNG, Geneva. Occurrence dataset

https://doi.org/10.15468/lwu4fj accessed via GBIF.org on 2018-08-29..

Accessed from R via rgbif (https://github.com/ropensci/rgbif) on

2018-08-29

Rights: The use of the data is allowed only for non-profit scientific use

and for non-profit nature conservation purpose.

[[21]]

<<rgbif citation>>

Citation: Cook J (2018). MSB Mammal Collection (Arctos). Version 35.17.

Museum of Southwestern Biology. Occurrence dataset

https://doi.org/10.15468/oirgxw accessed via GBIF.org on 2018-08-29..

Accessed from R via rgbif (https://github.com/ropensci/rgbif) on

2018-08-29

Rights:

[[22]]

<<rgbif citation>>

Citation: Royal Belgian Institute of Natural Sciences (2017). RBINS DaRWIN.

Occurrence dataset https://doi.org/10.15468/qxy4mc accessed via

GBIF.org on 2018-08-29.. Accessed from R via rgbif

(https://github.com/ropensci/rgbif) on 2018-08-29

Rights:

[[23]]

<<rgbif citation>>

Citation: Feeney R (2018). LACM Vertebrate Collection. Version 18.2. Natural

History Museum of Los Angeles County. Occurrence dataset

https://doi.org/10.15468/77rmwd accessed via GBIF.org on 2018-08-29..

Accessed from R via rgbif (https://github.com/ropensci/rgbif) on

2018-08-29

Rights:

[[24]]

<<rgbif citation>>

Citation: Slade N (2018). KUBI Mammalogy Collection. Version 26.13.

University of Kansas Biodiversity Institute. Occurrence dataset

https://doi.org/10.15468/a3woj7 accessed via GBIF.org on 2018-08-29..

Accessed from R via rgbif (https://github.com/ropensci/rgbif) on

2018-08-29

Rights:

[[25]]

<<rgbif citation>>

Citation: Tushabe H (2018). National Biodiversity Data Bank. Observation

records, 1900-2014. Version 1.2. National Biodiversity Data Bank.

Occurrence dataset https://doi.org/10.15468/djzgie accessed via

GBIF.org on 2018-08-29.. Accessed from R via rgbif

(https://github.com/ropensci/rgbif) on 2018-08-29

Rights:

[[26]]

<<rgbif citation>>

Citation: Marine Science Institute, UCSB. Paleobiology Database. Occurrence

dataset https://doi.org/10.15468/2durgn accessed via GBIF.org on

2018-08-29.. Accessed from R via rgbif

(https://github.com/ropensci/rgbif) on 2018-08-29

Rights:

[[27]]

<<rgbif citation>>

Citation: Western Australian Museum (2017). Western Australian Museum

provider for OZCAM. Occurrence dataset https://doi.org/10.15468/5qt0dm

accessed via GBIF.org on 2018-08-29.. Accessed from R via rgbif

(https://github.com/ropensci/rgbif) on 2018-08-29

Rights:

**2.) *Epomophorus gambianus***

[[1]]

<<rgbif citation>>

Citation: naturgucker.de. naturgucker. Occurrence dataset

https://doi.org/10.15468/uc1apo accessed via GBIF.org on 2018-08-29..

Accessed from R via rgbif (https://github.com/ropensci/rgbif) on

2018-08-29

Rights:

[[2]]

<<rgbif citation>>

Citation: iNaturalist.org (2018). iNaturalist Research-grade Observations.

Occurrence dataset https://doi.org/10.15468/ab3s5x accessed via

GBIF.org on 2018-08-29.. Accessed from R via rgbif

(https://github.com/ropensci/rgbif) on 2018-08-29

Rights:

[[3]]

<<rgbif citation>>

Citation: Yêhouénou Tessi D R (2017). List of animalia, fungi and plant

species recorded through naturalist observations and research

activities in Benin. Data mobilized in the framework of a JRS

Biodiversity Foundation project of Benin. Laboratory of Forest Sciences

(University of Abomey-Calavi). Occurrence dataset

https://doi.org/10.15468/22kwre accessed via GBIF.org on 2018-08-29..

Accessed from R via rgbif (https://github.com/ropensci/rgbif) on

2018-08-29

Rights:

[[4]]

<<rgbif citation>>

Citation: KINGBO A, KIKI P P B (2016). Census of the animals of Benin.

Version 1.2. GBIF Benin. Occurrence dataset

https://doi.org/10.15468/h7rqo9 accessed via GBIF.org on 2018-08-29..

Accessed from R via rgbif (https://github.com/ropensci/rgbif) on

2018-08-29

Rights: Public Domain (CC0 1.0)

[[5]]

<<rgbif citation>>

Citation: Orrell T, Hollowell T (2018). NMNH Extant Specimen Records.

Version 1.19. National Museum of Natural History, Smithsonian

Institution. Occurrence dataset https://doi.org/10.15468/hnhrg3

accessed via GBIF.org on 2018-08-29.. Accessed from R via rgbif

(https://github.com/ropensci/rgbif) on 2018-08-29

Rights:

[[6]]

<<rgbif citation>>

Citation: MNHN - Museum national d'Histoire naturelle (2018). The mammals

collection (ZM) of the Muséum national d'Histoire naturelle (MNHN -

Paris). Version 43.77. Occurrence dataset

https://doi.org/10.15468/j0xw9i accessed via GBIF.org on 2018-08-29..

Accessed from R via rgbif (https://github.com/ropensci/rgbif) on

2018-08-29

Rights:

[[7]]

<<rgbif citation>>

Citation: Conservation International. Rapid Assessment Program (RAP)

Biodiversity Survey Database. Occurrence dataset

https://doi.org/10.15468/tsrjm0 accessed via GBIF.org on 2018-08-29..

Accessed from R via rgbif (https://github.com/ropensci/rgbif) on

2018-08-29

Rights:

[[8]]

<<rgbif citation>>

Citation: Prestridge H (2016). Biodiversity Research and Teaching

Collections - TCWC Vertebrates. Version 9.1. Texas A&M University

Biodiversity Research and Teaching Collections. Occurrence dataset

https://doi.org/10.15468/szomia accessed via GBIF.org on 2018-08-29..

Accessed from R via rgbif (https://github.com/ropensci/rgbif) on

2018-08-29

Rights:

[[9]]

<<rgbif citation>>

Citation: Muséum d'histoire naturelle de la Ville de Genève - MHNG. Mammals

housed at MHNG, Geneva. Occurrence dataset

https://doi.org/10.15468/lwu4fj accessed via GBIF.org on 2018-08-29..

Accessed from R via rgbif (https://github.com/ropensci/rgbif) on

2018-08-29

Rights: The use of the data is allowed only for non-profit scientific use

and for non-profit nature conservation purpose.

[[10]]

<<rgbif citation>>

Citation: Trombone T (2016). AMNH Mammal Collections. American Museum of

Natural History. Occurrence dataset https://doi.org/10.15468/wu3poe

accessed via GBIF.org on 2018-08-29.. Accessed from R via rgbif

(https://github.com/ropensci/rgbif) on 2018-08-29

Rights:

[[11]]

<<rgbif citation>>

Citation: Grant S, Ferguson A (2018). Field Museum of Natural History

(Zoology) Mammal Collection. Version 9.5. Field Museum. Occurrence

dataset https://doi.org/10.15468/n4zgxw accessed via GBIF.org on

2018-08-29.. Accessed from R via rgbif

(https://github.com/ropensci/rgbif) on 2018-08-29

Rights:

[[12]]

<<rgbif citation>>

Citation: Feeney R (2018). LACM Vertebrate Collection. Version 18.2. Natural

History Museum of Los Angeles County. Occurrence dataset

https://doi.org/10.15468/77rmwd accessed via GBIF.org on 2018-08-29..

Accessed from R via rgbif (https://github.com/ropensci/rgbif) on

2018-08-29

Rights:

[[13]]

<<rgbif citation>>

Citation: Conroy C (2018). MVZ Mammal Collection (Arctos). Version 35.17.

Museum of Vertebrate Zoology. Occurrence dataset

https://doi.org/10.15468/uwudf9 accessed via GBIF.org on 2018-08-29..

Accessed from R via rgbif (https://github.com/ropensci/rgbif) on

2018-08-29

Rights:

[[14]]

<<rgbif citation>>

Citation: Millen B, Lim B (2018). Mammalogy Collection - Royal Ontario

Museum. Version 11.5. Royal Ontario Museum. Occurrence dataset

https://doi.org/10.15468/2rlrvh accessed via GBIF.org on 2018-08-29..

Accessed from R via rgbif (https://github.com/ropensci/rgbif) on

2018-08-29

Rights:

[[15]]

<<rgbif citation>>

Citation: Slade N (2018). KUBI Mammalogy Collection. Version 26.13.

University of Kansas Biodiversity Institute. Occurrence dataset

https://doi.org/10.15468/a3woj7 accessed via GBIF.org on 2018-08-29..

Accessed from R via rgbif (https://github.com/ropensci/rgbif) on

2018-08-29

Rights:

[[16]]

<<rgbif citation>>

Citation: Royal Belgian Institute of Natural Sciences (2017). RBINS DaRWIN.

Occurrence dataset https://doi.org/10.15468/qxy4mc accessed via

GBIF.org on 2018-08-29.. Accessed from R via rgbif

(https://github.com/ropensci/rgbif) on 2018-08-29

Rights:

[[17]]

<<rgbif citation>>

Citation: Abraczinskas L (2016). MSU Mammalogy, Ornithology and Vertebrate

Paleontology Collections. Version 8.1. Michigan State University

Museum. Occurrence dataset https://doi.org/10.15468/xypr72 accessed via

GBIF.org on 2018-08-29.. Accessed from R via rgbif

(https://github.com/ropensci/rgbif) on 2018-08-29

Rights:

[[18]]

<<rgbif citation>>

Citation: Bucci M (2016). UAZ Mammals. Version 5.1. University of Arizona

Museum of Natural History. Occurrence dataset

https://doi.org/10.15468/2swesj accessed via GBIF.org on 2018-08-29..

Accessed from R via rgbif (https://github.com/ropensci/rgbif) on

2018-08-29

Rights:

[[19]]

<<rgbif citation>>

Citation: Thompson C (2016). UMMZ Mammal Collection. Version 8.1. University

of Michigan Museum of Zoology. Occurrence dataset

https://doi.org/10.15468/dx3rcj accessed via GBIF.org on 2018-08-29..

Accessed from R via rgbif (https://github.com/ropensci/rgbif) on

2018-08-29

Rights:

[[20]]

<<rgbif citation>>

Citation: Western Australian Museum (2017). Western Australian Museum

provider for OZCAM. Occurrence dataset https://doi.org/10.15468/5qt0dm

accessed via GBIF.org on 2018-08-29.. Accessed from R via rgbif

(https://github.com/ropensci/rgbif) on 2018-08-29

Rights:

[[21]]

<<rgbif citation>>

Citation: Khidas K, Shorthouse D (2018). Canadian Museum of Nature Mammal

Collection. Version 1.8. Canadian Museum of Nature. Occurrence dataset

https://doi.org/10.15468/okgapc accessed via GBIF.org on 2018-08-29..

Accessed from R via rgbif (https://github.com/ropensci/rgbif) on

2018-08-29

Rights:

[[22]]

<<rgbif citation>>

Citation: Natural History Museum, University of Oslo (2018). Mammal

collection, Natural History Museum, University of Oslo. Version 31.137.

Occurrence dataset https://doi.org/10.15468/knvqao accessed via

GBIF.org on 2018-08-29.. Accessed from R via rgbif

(https://github.com/ropensci/rgbif) on 2018-08-29

Rights:

[[23]]

<<rgbif citation>>

Citation: Gall L (2018). Vertebrate Zoology Division - Mammalogy, Yale

Peabody Museum. Yale University Peabody Museum. Occurrence dataset

https://doi.org/10.15468/4mm6uc accessed via GBIF.org on 2018-08-29..

Accessed from R via rgbif (https://github.com/ropensci/rgbif) on

2018-08-29

Rights:

[[24]]

<<rgbif citation>>

Citation: Tushabe H (2018). National Biodiversity Data Bank. Observation

records, 1900-2014. Version 1.2. National Biodiversity Data Bank.

Occurrence dataset https://doi.org/10.15468/djzgie accessed via

GBIF.org on 2018-08-29.. Accessed from R via rgbif

(https://github.com/ropensci/rgbif) on 2018-08-29

Rights:

[[25]]

<<rgbif citation>>

Citation: Turpel A, Walisch T (2018). Collections and observation data

National Museum of Natural History Luxembourg. Musée national

d'histoire naturelle Luxembourg. Occurrence dataset

https://doi.org/10.15468/s2iu7d accessed via GBIF.org on 2018-08-29..

Accessed from R via rgbif (https://github.com/ropensci/rgbif) on

2018-08-29

Rights:

**3.) *Epomops franqueti***

[[1]]

<<rgbif citation>>

Citation: iNaturalist.org (2018). iNaturalist Research-grade Observations.

Occurrence dataset https://doi.org/10.15468/ab3s5x accessed via

GBIF.org on 2018-08-29.. Accessed from R via rgbif

(https://github.com/ropensci/rgbif) on 2018-08-29

Rights:

[[2]]

<<rgbif citation>>

Citation: Grant S, Ferguson A (2018). Field Museum of Natural History

(Zoology) Mammal Collection. Version 9.5. Field Museum. Occurrence

dataset https://doi.org/10.15468/n4zgxw accessed via GBIF.org on

2018-08-29.. Accessed from R via rgbif

(https://github.com/ropensci/rgbif) on 2018-08-29

Rights:

[[3]]

<<rgbif citation>>

Citation: KINGBO A, KIKI P P B (2016). Census of the animals of Benin.

Version 1.2. GBIF Benin. Occurrence dataset

https://doi.org/10.15468/h7rqo9 accessed via GBIF.org on 2018-08-29..

Accessed from R via rgbif (https://github.com/ropensci/rgbif) on

2018-08-29

Rights: Public Domain (CC0 1.0)

[[4]]

<<rgbif citation>>

Citation: Orrell T, Hollowell T (2018). NMNH Extant Specimen Records.

Version 1.19. National Museum of Natural History, Smithsonian

Institution. Occurrence dataset https://doi.org/10.15468/hnhrg3

accessed via GBIF.org on 2018-08-29.. Accessed from R via rgbif

(https://github.com/ropensci/rgbif) on 2018-08-29

Rights:

[[5]]

<<rgbif citation>>

Citation: MNHN - Museum national d'Histoire naturelle (2018). The mammals

collection (ZM) of the Muséum national d'Histoire naturelle (MNHN -

Paris). Version 43.77. Occurrence dataset

https://doi.org/10.15468/j0xw9i accessed via GBIF.org on 2018-08-29..

Accessed from R via rgbif (https://github.com/ropensci/rgbif) on

2018-08-29

Rights:

[[6]]

<<rgbif citation>>

Citation: Kiki P, Ganglo J (2017). Census of the threatened species of

Benin.. Version 1.5. GBIF Benin. Occurrence dataset

https://doi.org/10.15468/fbbbfl accessed via GBIF.org on 2018-08-29..

Accessed from R via rgbif (https://github.com/ropensci/rgbif) on

2018-08-29

Rights:

[[7]]

<<rgbif citation>>

Citation: Yêhouénou Tessi D R (2017). List of animalia, fungi and plant

species recorded through naturalist observations and research

activities in Benin. Data mobilized in the framework of a JRS

Biodiversity Foundation project of Benin. Laboratory of Forest Sciences

(University of Abomey-Calavi). Occurrence dataset

https://doi.org/10.15468/22kwre accessed via GBIF.org on 2018-08-29..

Accessed from R via rgbif (https://github.com/ropensci/rgbif) on

2018-08-29

Rights:

[[8]]

<<rgbif citation>>

Citation: Cook J (2018). MSB Mammal Collection (Arctos). Version 35.17.

Museum of Southwestern Biology. Occurrence dataset

https://doi.org/10.15468/oirgxw accessed via GBIF.org on 2018-08-29..

Accessed from R via rgbif (https://github.com/ropensci/rgbif) on

2018-08-29

Rights:

[[9]]

<<rgbif citation>>

Citation: Tushabe H (2018). National Biodiversity Data Bank. Observation

records, 1900-2014. Version 1.2. National Biodiversity Data Bank.

Occurrence dataset https://doi.org/10.15468/djzgie accessed via

GBIF.org on 2018-08-29.. Accessed from R via rgbif

(https://github.com/ropensci/rgbif) on 2018-08-29

Rights:

[[10]]

<<rgbif citation>>

Citation: Biological Station of Doñana (CSIC) (2018). Estacion Biologica

Donana - CSIC, Mammal Collection. Occurrence dataset

https://doi.org/10.15468/fwrsxr accessed via GBIF.org on 2018-08-29..

Accessed from R via rgbif (https://github.com/ropensci/rgbif) on

2018-08-29

Rights:

[[11]]

<<rgbif citation>>

Citation: Muséum d'histoire naturelle de la Ville de Genève - MHNG. Mammals

housed at MHNG, Geneva. Occurrence dataset

https://doi.org/10.15468/lwu4fj accessed via GBIF.org on 2018-08-29..

Accessed from R via rgbif (https://github.com/ropensci/rgbif) on

2018-08-29

Rights: The use of the data is allowed only for non-profit scientific use

and for non-profit nature conservation purpose.

[[12]]

<<rgbif citation>>

Citation: Millen B, Lim B (2018). Mammalogy Collection - Royal Ontario

Museum. Version 11.5. Royal Ontario Museum. Occurrence dataset

https://doi.org/10.15468/2rlrvh accessed via GBIF.org on 2018-08-29..

Accessed from R via rgbif (https://github.com/ropensci/rgbif) on

2018-08-29

Rights:

[[13]]

<<rgbif citation>>

Citation: Feeney R (2018). LACM Vertebrate Collection. Version 18.2. Natural

History Museum of Los Angeles County. Occurrence dataset

https://doi.org/10.15468/77rmwd accessed via GBIF.org on 2018-08-29..

Accessed from R via rgbif (https://github.com/ropensci/rgbif) on

2018-08-29

Rights:

[[14]]

<<rgbif citation>>

Citation: Thompson C (2016). UMMZ Mammal Collection. Version 8.1. University

of Michigan Museum of Zoology. Occurrence dataset

https://doi.org/10.15468/dx3rcj accessed via GBIF.org on 2018-08-29..

Accessed from R via rgbif (https://github.com/ropensci/rgbif) on

2018-08-29

Rights:

[[15]]

<<rgbif citation>>

Citation: Trombone T (2016). AMNH Mammal Collections. American Museum of

Natural History. Occurrence dataset https://doi.org/10.15468/wu3poe

accessed via GBIF.org on 2018-08-29.. Accessed from R via rgbif

(https://github.com/ropensci/rgbif) on 2018-08-29

Rights:

[[16]]

<<rgbif citation>>

Citation: Mateke C, Mulwanda S, Nachinga D (2018). Livingstone Museum Bat

Collection. Livingstone Museum. Occurrence dataset

https://doi.org/10.15468/i6cor7 accessed via GBIF.org on 2018-08-29..

Accessed from R via rgbif (https://github.com/ropensci/rgbif) on

2018-08-29

Rights:

[[17]]

<<rgbif citation>>

Citation: Dillman C, Dardia C (2018). CUMV Mammal Collection. Version 27.17.

Cornell University Museum of Vertebrates. Occurrence dataset

https://doi.org/10.15468/kkgkne accessed via GBIF.org on 2018-08-29..

Accessed from R via rgbif (https://github.com/ropensci/rgbif) on

2018-08-29

Rights:

[[18]]

<<rgbif citation>>

Citation: Scholes III, Ph.D. E (2015). Macaulay Library Audio and Video

Collection. Cornell Lab of Ornithology. Occurrence dataset

https://doi.org/10.15468/ckcdpy accessed via GBIF.org on 2018-08-29..

Accessed from R via rgbif (https://github.com/ropensci/rgbif) on

2018-08-29

Rights: http://creativecommons.org/publicdomain/zero/1.0 and

http://vertnet.org/resources/norms.html

[[19]]

<<rgbif citation>>

Citation: Garner H (2016). TTU Mammals Collection. Version 9.1. Museum of

Texas Tech University (TTU). Occurrence dataset

https://doi.org/10.15468/yocqyp accessed via GBIF.org on 2018-08-29..

Accessed from R via rgbif (https://github.com/ropensci/rgbif) on

2018-08-29

Rights:

[[20]]

<<rgbif citation>>

Citation: Bradley J (2018). UWBM Mammalogy Collection (Arctos). University

of Washington Burke Museum. Occurrence dataset

https://doi.org/10.15468/qziy3w accessed via GBIF.org on 2018-08-29..

Accessed from R via rgbif (https://github.com/ropensci/rgbif) on

2018-08-29

Rights:

[[21]]

<<rgbif citation>>

Citation: Conroy C (2018). MVZ Mammal Collection (Arctos). Version 35.17.

Museum of Vertebrate Zoology. Occurrence dataset

https://doi.org/10.15468/uwudf9 accessed via GBIF.org on 2018-08-29..

Accessed from R via rgbif (https://github.com/ropensci/rgbif) on

2018-08-29

Rights:

[[22]]

<<rgbif citation>>

Citation: Royal Belgian Institute of Natural Sciences (2017). RBINS DaRWIN.

Occurrence dataset https://doi.org/10.15468/qxy4mc accessed via

GBIF.org on 2018-08-29.. Accessed from R via rgbif

(https://github.com/ropensci/rgbif) on 2018-08-29

Rights:

[[23]]

<<rgbif citation>>

Citation: Harvard University M, Morris P J (2018). Museum of Comparative

Zoology, Harvard University. Version 162.115. Museum of Comparative

Zoology, Harvard University. Occurrence dataset

https://doi.org/10.15468/p5rupv accessed via GBIF.org on 2018-08-29..

Accessed from R via rgbif (https://github.com/ropensci/rgbif) on

2018-08-29

Rights:

**4.) *Hypsignathus monstrosus***

[[1]]

<<rgbif citation>>

Citation: naturgucker.de. naturgucker. Occurrence dataset

https://doi.org/10.15468/uc1apo accessed via GBIF.org on 2018-08-29..

Accessed from R via rgbif (https://github.com/ropensci/rgbif) on

2018-08-29

Rights:

[[2]]

<<rgbif citation>>

Citation: iNaturalist.org (2018). iNaturalist Research-grade Observations.

Occurrence dataset https://doi.org/10.15468/ab3s5x accessed via

GBIF.org on 2018-08-29.. Accessed from R via rgbif

(https://github.com/ropensci/rgbif) on 2018-08-29

Rights:

[[3]]

<<rgbif citation>>

Citation: Grant S, Ferguson A (2018). Field Museum of Natural History

(Zoology) Mammal Collection. Version 9.5. Field Museum. Occurrence

dataset https://doi.org/10.15468/n4zgxw accessed via GBIF.org on

2018-08-29.. Accessed from R via rgbif

(https://github.com/ropensci/rgbif) on 2018-08-29

Rights:

[[4]]

<<rgbif citation>>

Citation: KINGBO A, KIKI P P B (2016). Census of the animals of Benin.

Version 1.2. GBIF Benin. Occurrence dataset

https://doi.org/10.15468/h7rqo9 accessed via GBIF.org on 2018-08-29..

Accessed from R via rgbif (https://github.com/ropensci/rgbif) on

2018-08-29

Rights: Public Domain (CC0 1.0)

[[5]]

<<rgbif citation>>

Citation: MNHN - Museum national d'Histoire naturelle (2018). The mammals

collection (ZM) of the Muséum national d'Histoire naturelle (MNHN -

Paris). Version 43.77. Occurrence dataset

https://doi.org/10.15468/j0xw9i accessed via GBIF.org on 2018-08-29..

Accessed from R via rgbif (https://github.com/ropensci/rgbif) on

2018-08-29

Rights:

[[6]]

<<rgbif citation>>

Citation: Orrell T, Hollowell T (2018). NMNH Extant Specimen Records.

Version 1.19. National Museum of Natural History, Smithsonian

Institution. Occurrence dataset https://doi.org/10.15468/hnhrg3

accessed via GBIF.org on 2018-08-29.. Accessed from R via rgbif

(https://github.com/ropensci/rgbif) on 2018-08-29

Rights:

[[7]]

<<rgbif citation>>

Citation: Kiki P, Ganglo J (2017). Census of the threatened species of

Benin.. Version 1.5. GBIF Benin. Occurrence dataset

https://doi.org/10.15468/fbbbfl accessed via GBIF.org on 2018-08-29..

Accessed from R via rgbif (https://github.com/ropensci/rgbif) on

2018-08-29

Rights:

[[8]]

<<rgbif citation>>

Citation: Conservation International. Rapid Assessment Program (RAP)

Biodiversity Survey Database. Occurrence dataset

https://doi.org/10.15468/tsrjm0 accessed via GBIF.org on 2018-08-29..

Accessed from R via rgbif (https://github.com/ropensci/rgbif) on

2018-08-29

Rights:

[[9]]

<<rgbif citation>>

Citation: Tushabe H (2018). National Biodiversity Data Bank. Observation

records, 1900-2014. Version 1.2. National Biodiversity Data Bank.

Occurrence dataset https://doi.org/10.15468/djzgie accessed via

GBIF.org on 2018-08-29.. Accessed from R via rgbif

(https://github.com/ropensci/rgbif) on 2018-08-29

Rights:

[[10]]

<<rgbif citation>>

Citation: Muséum d'histoire naturelle de la Ville de Genève - MHNG. Mammals

housed at MHNG, Geneva. Occurrence dataset

https://doi.org/10.15468/lwu4fj accessed via GBIF.org on 2018-08-29..

Accessed from R via rgbif (https://github.com/ropensci/rgbif) on

2018-08-29

Rights: The use of the data is allowed only for non-profit scientific use

and for non-profit nature conservation purpose.

[[11]]

<<rgbif citation>>

Citation: Trombone T (2016). AMNH Mammal Collections. American Museum of

Natural History. Occurrence dataset https://doi.org/10.15468/wu3poe

accessed via GBIF.org on 2018-08-29.. Accessed from R via rgbif

(https://github.com/ropensci/rgbif) on 2018-08-29

Rights:

[[12]]

<<rgbif citation>>

Citation: Scholes III, Ph.D. E (2015). Macaulay Library Audio and Video

Collection. Cornell Lab of Ornithology. Occurrence dataset

https://doi.org/10.15468/ckcdpy accessed via GBIF.org on 2018-08-29..

Accessed from R via rgbif (https://github.com/ropensci/rgbif) on

2018-08-29

Rights: http://creativecommons.org/publicdomain/zero/1.0 and

http://vertnet.org/resources/norms.html

[[13]]

<<rgbif citation>>

Citation: Millen B, Lim B (2018). Mammalogy Collection - Royal Ontario

Museum. Version 11.5. Royal Ontario Museum. Occurrence dataset

https://doi.org/10.15468/2rlrvh accessed via GBIF.org on 2018-08-29..

Accessed from R via rgbif (https://github.com/ropensci/rgbif) on

2018-08-29

Rights:

[[14]]

<<rgbif citation>>

Citation: Dillman C, Dardia C (2018). CUMV Mammal Collection. Version 27.17.

Cornell University Museum of Vertebrates. Occurrence dataset

https://doi.org/10.15468/kkgkne accessed via GBIF.org on 2018-08-29..

Accessed from R via rgbif (https://github.com/ropensci/rgbif) on

2018-08-29

Rights:

[[15]]

<<rgbif citation>>

Citation: Feeney R (2018). LACM Vertebrate Collection. Version 18.2. Natural

History Museum of Los Angeles County. Occurrence dataset

https://doi.org/10.15468/77rmwd accessed via GBIF.org on 2018-08-29..

Accessed from R via rgbif (https://github.com/ropensci/rgbif) on

2018-08-29

Rights:

[[16]]

<<rgbif citation>>

Citation: Royal Belgian Institute of Natural Sciences (2017). RBINS DaRWIN.

Occurrence dataset https://doi.org/10.15468/qxy4mc accessed via

GBIF.org on 2018-08-29.. Accessed from R via rgbif

(https://github.com/ropensci/rgbif) on 2018-08-29

Rights:

[[17]]

<<rgbif citation>>

Citation: Cook J (2018). MSB Mammal Collection (Arctos). Version 35.17.

Museum of Southwestern Biology. Occurrence dataset

https://doi.org/10.15468/oirgxw accessed via GBIF.org on 2018-08-29..

Accessed from R via rgbif (https://github.com/ropensci/rgbif) on

2018-08-29

Rights:

[[18]]

<<rgbif citation>>

Citation: Slade N (2018). KUBI Mammalogy Collection. Version 26.13.

University of Kansas Biodiversity Institute. Occurrence dataset

https://doi.org/10.15468/a3woj7 accessed via GBIF.org on 2018-08-29..

Accessed from R via rgbif (https://github.com/ropensci/rgbif) on

2018-08-29

Rights:

[[19]]

<<rgbif citation>>

Citation: Australian Museum (2017). Australian Museum provider for OZCAM.

Occurrence dataset https://doi.org/10.15468/e7susi accessed via

GBIF.org on 2018-08-29.. Accessed from R via rgbif

(https://github.com/ropensci/rgbif) on 2018-08-29

Rights:

**5.) *Micropteropus pusillus***

[[1]]

<<rgbif citation>>

Citation: iNaturalist.org (2018). iNaturalist Research-grade Observations.

Occurrence dataset https://doi.org/10.15468/ab3s5x accessed via

GBIF.org on 2018-08-29.. Accessed from R via rgbif

(https://github.com/ropensci/rgbif) on 2018-08-29

Rights:

[[2]]

<<rgbif citation>>

Citation: Orrell T, Hollowell T (2018). NMNH Extant Specimen Records.

Version 1.19. National Museum of Natural History, Smithsonian

Institution. Occurrence dataset https://doi.org/10.15468/hnhrg3

accessed via GBIF.org on 2018-08-29.. Accessed from R via rgbif

(https://github.com/ropensci/rgbif) on 2018-08-29

Rights:

[[3]]

<<rgbif citation>>

Citation: Grant S, Ferguson A (2018). Field Museum of Natural History

(Zoology) Mammal Collection. Version 9.5. Field Museum. Occurrence

dataset https://doi.org/10.15468/n4zgxw accessed via GBIF.org on

2018-08-29.. Accessed from R via rgbif

(https://github.com/ropensci/rgbif) on 2018-08-29

Rights:

[[4]]

<<rgbif citation>>

Citation: KINGBO A, KIKI P P B (2016). Census of the animals of Benin.

Version 1.2. GBIF Benin. Occurrence dataset

https://doi.org/10.15468/h7rqo9 accessed via GBIF.org on 2018-08-29..

Accessed from R via rgbif (https://github.com/ropensci/rgbif) on

2018-08-29

Rights: Public Domain (CC0 1.0)

[[5]]

<<rgbif citation>>

Citation: MNHN - Museum national d'Histoire naturelle (2018). The mammals

collection (ZM) of the Muséum national d'Histoire naturelle (MNHN -

Paris). Version 43.77. Occurrence dataset

https://doi.org/10.15468/j0xw9i accessed via GBIF.org on 2018-08-29..

Accessed from R via rgbif (https://github.com/ropensci/rgbif) on

2018-08-29

Rights:

[[6]]

<<rgbif citation>>

Citation: Gall L (2018). Vertebrate Zoology Division - Mammalogy, Yale

Peabody Museum. Yale University Peabody Museum. Occurrence dataset

https://doi.org/10.15468/4mm6uc accessed via GBIF.org on 2018-08-29..

Accessed from R via rgbif (https://github.com/ropensci/rgbif) on

2018-08-29

Rights:

[[7]]

<<rgbif citation>>

Citation: Conservation International. Rapid Assessment Program (RAP)

Biodiversity Survey Database. Occurrence dataset

https://doi.org/10.15468/tsrjm0 accessed via GBIF.org on 2018-08-29..

Accessed from R via rgbif (https://github.com/ropensci/rgbif) on

2018-08-29

Rights:

[[8]]

<<rgbif citation>>

Citation: Prestridge H (2016). Biodiversity Research and Teaching

Collections - TCWC Vertebrates. Version 9.1. Texas A&M University

Biodiversity Research and Teaching Collections. Occurrence dataset

https://doi.org/10.15468/szomia accessed via GBIF.org on 2018-08-29..

Accessed from R via rgbif (https://github.com/ropensci/rgbif) on

2018-08-29

Rights:

[[9]]

<<rgbif citation>>

Citation: Tushabe H (2018). National Biodiversity Data Bank. Observation

records, 1900-2014. Version 1.2. National Biodiversity Data Bank.

Occurrence dataset https://doi.org/10.15468/djzgie accessed via

GBIF.org on 2018-08-29.. Accessed from R via rgbif

(https://github.com/ropensci/rgbif) on 2018-08-29

Rights:

[[10]]

<<rgbif citation>>

Citation: Cook J (2018). MSB Mammal Collection (Arctos). Version 35.17.

Museum of Southwestern Biology. Occurrence dataset

https://doi.org/10.15468/oirgxw accessed via GBIF.org on 2018-08-29..

Accessed from R via rgbif (https://github.com/ropensci/rgbif) on

2018-08-29

Rights:

[[11]]

<<rgbif citation>>

Citation: Muséum d'histoire naturelle de la Ville de Genève - MHNG. Mammals

housed at MHNG, Geneva. Occurrence dataset

https://doi.org/10.15468/lwu4fj accessed via GBIF.org on 2018-08-29..

Accessed from R via rgbif (https://github.com/ropensci/rgbif) on

2018-08-29

Rights: The use of the data is allowed only for non-profit scientific use

and for non-profit nature conservation purpose.

[[12]]

<<rgbif citation>>

Citation: Abraczinskas L (2016). MSU Mammalogy, Ornithology and Vertebrate

Paleontology Collections. Version 8.1. Michigan State University

Museum. Occurrence dataset https://doi.org/10.15468/xypr72 accessed via

GBIF.org on 2018-08-29.. Accessed from R via rgbif

(https://github.com/ropensci/rgbif) on 2018-08-29

Rights:

[[13]]

<<rgbif citation>>

Citation: Feeney R (2018). LACM Vertebrate Collection. Version 18.2. Natural

History Museum of Los Angeles County. Occurrence dataset

https://doi.org/10.15468/77rmwd accessed via GBIF.org on 2018-08-29..

Accessed from R via rgbif (https://github.com/ropensci/rgbif) on

2018-08-29

Rights:

[[14]]

<<rgbif citation>>

Citation: Millen B, Lim B (2018). Mammalogy Collection - Royal Ontario

Museum. Version 11.5. Royal Ontario Museum. Occurrence dataset

https://doi.org/10.15468/2rlrvh accessed via GBIF.org on 2018-08-29..

Accessed from R via rgbif (https://github.com/ropensci/rgbif) on

2018-08-29

Rights:

[[15]]

<<rgbif citation>>

Citation: Thompson C (2016). UMMZ Mammal Collection. Version 8.1. University

of Michigan Museum of Zoology. Occurrence dataset

https://doi.org/10.15468/dx3rcj accessed via GBIF.org on 2018-08-29..

Accessed from R via rgbif (https://github.com/ropensci/rgbif) on

2018-08-29

Rights:

[[16]]

<<rgbif citation>>

Citation: Trombone T (2016). AMNH Mammal Collections. American Museum of

Natural History. Occurrence dataset https://doi.org/10.15468/wu3poe

accessed via GBIF.org on 2018-08-29.. Accessed from R via rgbif

(https://github.com/ropensci/rgbif) on 2018-08-29

Rights:

[[17]]

<<rgbif citation>>

Citation: Mateke C, Mulwanda S, Nachinga D (2018). Livingstone Museum Bat

Collection. Livingstone Museum. Occurrence dataset

https://doi.org/10.15468/i6cor7 accessed via GBIF.org on 2018-08-29..

Accessed from R via rgbif (https://github.com/ropensci/rgbif) on

2018-08-29

Rights:

[[18]]

<<rgbif citation>>

Citation: Garner H (2016). TTU Mammals Collection. Version 9.1. Museum of

Texas Tech University (TTU). Occurrence dataset

https://doi.org/10.15468/yocqyp accessed via GBIF.org on 2018-08-29..

Accessed from R via rgbif (https://github.com/ropensci/rgbif) on

2018-08-29

Rights:

[[19]]

<<rgbif citation>>

Citation: Flannery M, Fong J (2018). CAS Mammalogy (MAM). Version 133.136.

California Academy of Sciences. Occurrence dataset

https://doi.org/10.15468/dhbozg accessed via GBIF.org on 2018-08-29..

Accessed from R via rgbif (https://github.com/ropensci/rgbif) on

2018-08-29

Rights:

[[20]]

<<rgbif citation>>

Citation: Bucci M (2016). UAZ Mammals. Version 5.1. University of Arizona

Museum of Natural History. Occurrence dataset

https://doi.org/10.15468/2swesj accessed via GBIF.org on 2018-08-29..

Accessed from R via rgbif (https://github.com/ropensci/rgbif) on

2018-08-29

Rights:

[[21]]

<<rgbif citation>>

Citation: Conroy C (2018). MVZ Mammal Collection (Arctos). Version 35.17.

Museum of Vertebrate Zoology. Occurrence dataset

https://doi.org/10.15468/uwudf9 accessed via GBIF.org on 2018-08-29..

Accessed from R via rgbif (https://github.com/ropensci/rgbif) on

2018-08-29

Rights:

[[22]]

<<rgbif citation>>

Citation: Slade N (2018). KUBI Mammalogy Collection. Version 26.13.

University of Kansas Biodiversity Institute. Occurrence dataset

https://doi.org/10.15468/a3woj7 accessed via GBIF.org on 2018-08-29..

Accessed from R via rgbif (https://github.com/ropensci/rgbif) on

2018-08-29

Rights:

[[23]]

<<rgbif citation>>

Citation: Royal Belgian Institute of Natural Sciences (2017). RBINS DaRWIN.

Occurrence dataset https://doi.org/10.15468/qxy4mc accessed via

GBIF.org on 2018-08-29.. Accessed from R via rgbif

(https://github.com/ropensci/rgbif) on 2018-08-29

Rights:

[[24]]

<<rgbif citation>>

Citation: Western Australian Museum (2017). Western Australian Museum

provider for OZCAM. Occurrence dataset https://doi.org/10.15468/5qt0dm

accessed via GBIF.org on 2018-08-29.. Accessed from R via rgbif

(https://github.com/ropensci/rgbif) on 2018-08-29

Rights:

**6.) *Mops condylurus***

[[1]]

<<rgbif citation>>

Citation: iNaturalist.org (2018). iNaturalist Research-grade Observations.

Occurrence dataset https://doi.org/10.15468/ab3s5x accessed via

GBIF.org on 2018-08-29.. Accessed from R via rgbif

(https://github.com/ropensci/rgbif) on 2018-08-29

Rights:

[[2]]

<<rgbif citation>>

Citation: Grant S, Ferguson A (2018). Field Museum of Natural History

(Zoology) Mammal Collection. Version 9.5. Field Museum. Occurrence

dataset https://doi.org/10.15468/n4zgxw accessed via GBIF.org on

2018-08-29.. Accessed from R via rgbif

(https://github.com/ropensci/rgbif) on 2018-08-29

Rights:

[[3]]

<<rgbif citation>>

Citation: KINGBO A, KIKI P P B (2016). Census of the animals of Benin.

Version 1.2. GBIF Benin. Occurrence dataset

https://doi.org/10.15468/h7rqo9 accessed via GBIF.org on 2018-08-29..

Accessed from R via rgbif (https://github.com/ropensci/rgbif) on

2018-08-29

Rights: Public Domain (CC0 1.0)

[[4]]

<<rgbif citation>>

Citation: MNHN - Museum national d'Histoire naturelle (2018). The mammals

collection (ZM) of the Muséum national d'Histoire naturelle (MNHN -

Paris). Version 43.77. Occurrence dataset

https://doi.org/10.15468/j0xw9i accessed via GBIF.org on 2018-08-29..

Accessed from R via rgbif (https://github.com/ropensci/rgbif) on

2018-08-29

Rights:

[[5]]

<<rgbif citation>>

Citation: Mateke C, Mulwanda S, Nachinga D (2018). Livingstone Museum Bat

Collection. Livingstone Museum. Occurrence dataset

https://doi.org/10.15468/i6cor7 accessed via GBIF.org on 2018-08-29..

Accessed from R via rgbif (https://github.com/ropensci/rgbif) on

2018-08-29

Rights:

[[6]]

<<rgbif citation>>

Citation: Cook J (2018). MSB Mammal Collection (Arctos). Version 35.17.

Museum of Southwestern Biology. Occurrence dataset

https://doi.org/10.15468/oirgxw accessed via GBIF.org on 2018-08-29..

Accessed from R via rgbif (https://github.com/ropensci/rgbif) on

2018-08-29

Rights:

[[7]]

<<rgbif citation>>

Citation: Orrell T, Hollowell T (2018). NMNH Extant Specimen Records.

Version 1.19. National Museum of Natural History, Smithsonian

Institution. Occurrence dataset https://doi.org/10.15468/hnhrg3

accessed via GBIF.org on 2018-08-29.. Accessed from R via rgbif

(https://github.com/ropensci/rgbif) on 2018-08-29

Rights:

[[8]]

<<rgbif citation>>

Citation: Muséum d'histoire naturelle de la Ville de Genève - MHNG. Mammals

housed at MHNG, Geneva. Occurrence dataset

https://doi.org/10.15468/lwu4fj accessed via GBIF.org on 2018-08-29..

Accessed from R via rgbif (https://github.com/ropensci/rgbif) on

2018-08-29

Rights: The use of the data is allowed only for non-profit scientific use

and for non-profit nature conservation purpose.

[[9]]

<<rgbif citation>>

Citation: Feeney R (2018). LACM Vertebrate Collection. Version 18.2. Natural

History Museum of Los Angeles County. Occurrence dataset

https://doi.org/10.15468/77rmwd accessed via GBIF.org on 2018-08-29..

Accessed from R via rgbif (https://github.com/ropensci/rgbif) on

2018-08-29

Rights:

[[10]]

<<rgbif citation>>

Citation: Abraczinskas L (2016). MSU Mammalogy, Ornithology and Vertebrate

Paleontology Collections. Version 8.1. Michigan State University

Museum. Occurrence dataset https://doi.org/10.15468/xypr72 accessed via

GBIF.org on 2018-08-29.. Accessed from R via rgbif

(https://github.com/ropensci/rgbif) on 2018-08-29

Rights:

[[11]]

<<rgbif citation>>

Citation: Conroy C (2018). MVZ Mammal Collection (Arctos). Version 35.17.

Museum of Vertebrate Zoology. Occurrence dataset

https://doi.org/10.15468/uwudf9 accessed via GBIF.org on 2018-08-29..

Accessed from R via rgbif (https://github.com/ropensci/rgbif) on

2018-08-29

Rights:

[[12]]

<<rgbif citation>>

Citation: Slade N (2018). KUBI Mammalogy Collection. Version 26.13.

University of Kansas Biodiversity Institute. Occurrence dataset

https://doi.org/10.15468/a3woj7 accessed via GBIF.org on 2018-08-29..

Accessed from R via rgbif (https://github.com/ropensci/rgbif) on

2018-08-29

Rights:

[[13]]

<<rgbif citation>>

Citation: Flannery M, Fong J (2018). CAS Mammalogy (MAM). Version 133.136.

California Academy of Sciences. Occurrence dataset

https://doi.org/10.15468/dhbozg accessed via GBIF.org on 2018-08-29..

Accessed from R via rgbif (https://github.com/ropensci/rgbif) on

2018-08-29

Rights:

[[14]]

<<rgbif citation>>

Citation: Royal Belgian Institute of Natural Sciences (2017). RBINS DaRWIN.

Occurrence dataset https://doi.org/10.15468/qxy4mc accessed via

GBIF.org on 2018-08-29.. Accessed from R via rgbif

(https://github.com/ropensci/rgbif) on 2018-08-29

Rights:

[[15]]

<<rgbif citation>>

Citation: Gall L (2018). Vertebrate Zoology Division - Mammalogy, Yale

Peabody Museum. Yale University Peabody Museum. Occurrence dataset

https://doi.org/10.15468/4mm6uc accessed via GBIF.org on 2018-08-29..

Accessed from R via rgbif (https://github.com/ropensci/rgbif) on

2018-08-29

Rights:

[[16]]

<<rgbif citation>>

Citation: Bucci M (2016). UAZ Mammals. Version 5.1. University of Arizona

Museum of Natural History. Occurrence dataset

https://doi.org/10.15468/2swesj accessed via GBIF.org on 2018-08-29..

Accessed from R via rgbif (https://github.com/ropensci/rgbif) on

2018-08-29

Rights:

[[17]]

<<rgbif citation>>

Citation: Western Australian Museum (2017). Western Australian Museum

provider for OZCAM. Occurrence dataset https://doi.org/10.15468/5qt0dm

accessed via GBIF.org on 2018-08-29.. Accessed from R via rgbif

(https://github.com/ropensci/rgbif) on 2018-08-29

Rights:

[[18]]

<<rgbif citation>>

Citation: Tushabe H (2018). National Biodiversity Data Bank. Observation

records, 1900-2014. Version 1.2. National Biodiversity Data Bank.

Occurrence dataset https://doi.org/10.15468/djzgie accessed via

GBIF.org on 2018-08-29.. Accessed from R via rgbif

(https://github.com/ropensci/rgbif) on 2018-08-29

Rights:

**7.) *Myonycteris torquata***

[[1]]

<<rgbif citation>>

Citation: iNaturalist.org (2018). iNaturalist Research-grade Observations.

Occurrence dataset https://doi.org/10.15468/ab3s5x accessed via

GBIF.org on 2018-08-29.. Accessed from R via rgbif

(https://github.com/ropensci/rgbif) on 2018-08-29

Rights:

[[2]]

<<rgbif citation>>

Citation: Grant S, Ferguson A (2018). Field Museum of Natural History

(Zoology) Mammal Collection. Version 9.5. Field Museum. Occurrence

dataset https://doi.org/10.15468/n4zgxw accessed via GBIF.org on

2018-08-29.. Accessed from R via rgbif

(https://github.com/ropensci/rgbif) on 2018-08-29

Rights:

[[3]]

<<rgbif citation>>

Citation: KINGBO A, KIKI P P B (2016). Census of the animals of Benin.

Version 1.2. GBIF Benin. Occurrence dataset

https://doi.org/10.15468/h7rqo9 accessed via GBIF.org on 2018-08-29..

Accessed from R via rgbif (https://github.com/ropensci/rgbif) on

2018-08-29

Rights: Public Domain (CC0 1.0)

[[4]]

<<rgbif citation>>

Citation: MNHN - Museum national d'Histoire naturelle (2018). The mammals

collection (ZM) of the Muséum national d'Histoire naturelle (MNHN -

Paris). Version 43.77. Occurrence dataset

https://doi.org/10.15468/j0xw9i accessed via GBIF.org on 2018-08-29..

Accessed from R via rgbif (https://github.com/ropensci/rgbif) on

2018-08-29

Rights:

[[5]]

<<rgbif citation>>

Citation: Kiki P, Ganglo J (2017). Census of the threatened species of

Benin.. Version 1.5. GBIF Benin. Occurrence dataset

https://doi.org/10.15468/fbbbfl accessed via GBIF.org on 2018-08-29..

Accessed from R via rgbif (https://github.com/ropensci/rgbif) on

2018-08-29

Rights:

[[6]]

<<rgbif citation>>

Citation: Conservation International. Rapid Assessment Program (RAP)

Biodiversity Survey Database. Occurrence dataset

https://doi.org/10.15468/tsrjm0 accessed via GBIF.org on 2018-08-29..

Accessed from R via rgbif (https://github.com/ropensci/rgbif) on

2018-08-29

Rights:

[[7]]

<<rgbif citation>>

Citation: Orrell T, Hollowell T (2018). NMNH Extant Specimen Records.

Version 1.19. National Museum of Natural History, Smithsonian

Institution. Occurrence dataset https://doi.org/10.15468/hnhrg3

accessed via GBIF.org on 2018-08-29.. Accessed from R via rgbif

(https://github.com/ropensci/rgbif) on 2018-08-29

Rights:

[[8]]

<<rgbif citation>>

Citation: Tushabe H (2018). National Biodiversity Data Bank. Observation

records, 1900-2014. Version 1.2. National Biodiversity Data Bank.

Occurrence dataset https://doi.org/10.15468/djzgie accessed via

GBIF.org on 2018-08-29.. Accessed from R via rgbif

(https://github.com/ropensci/rgbif) on 2018-08-29

Rights:

[[9]]

<<rgbif citation>>

Citation: Trombone T (2016). AMNH Mammal Collections. American Museum of

Natural History. Occurrence dataset https://doi.org/10.15468/wu3poe

accessed via GBIF.org on 2018-08-29.. Accessed from R via rgbif

(https://github.com/ropensci/rgbif) on 2018-08-29

Rights:

[[10]]

<<rgbif citation>>

Citation: Biological Station of Doñana (CSIC) (2018). Estacion Biologica

Donana - CSIC, Mammal Collection. Occurrence dataset

https://doi.org/10.15468/fwrsxr accessed via GBIF.org on 2018-08-29..

Accessed from R via rgbif (https://github.com/ropensci/rgbif) on

2018-08-29

Rights:

[[11]]

<<rgbif citation>>

Citation: Muséum d'histoire naturelle de la Ville de Genève - MHNG. Mammals

housed at MHNG, Geneva. Occurrence dataset

https://doi.org/10.15468/lwu4fj accessed via GBIF.org on 2018-08-29..

Accessed from R via rgbif (https://github.com/ropensci/rgbif) on

2018-08-29

Rights: The use of the data is allowed only for non-profit scientific use

and for non-profit nature conservation purpose.

[[12]]

<<rgbif citation>>

Citation: Millen B, Lim B (2018). Mammalogy Collection - Royal Ontario

Museum. Version 11.5. Royal Ontario Museum. Occurrence dataset

https://doi.org/10.15468/2rlrvh accessed via GBIF.org on 2018-08-29..

Accessed from R via rgbif (https://github.com/ropensci/rgbif) on

2018-08-29

Rights:

[[13]]

<<rgbif citation>>

Citation: Garner H (2016). TTU Mammals Collection. Version 9.1. Museum of

Texas Tech University (TTU). Occurrence dataset

https://doi.org/10.15468/yocqyp accessed via GBIF.org on 2018-08-29..

Accessed from R via rgbif (https://github.com/ropensci/rgbif) on

2018-08-29

Rights:

[[14]]

<<rgbif citation>>

Citation: Feeney R (2018). LACM Vertebrate Collection. Version 18.2. Natural

History Museum of Los Angeles County. Occurrence dataset

https://doi.org/10.15468/77rmwd accessed via GBIF.org on 2018-08-29..

Accessed from R via rgbif (https://github.com/ropensci/rgbif) on

2018-08-29

Rights:

[[15]]

<<rgbif citation>>

Citation: Cook J (2018). MSB Mammal Collection (Arctos). Version 35.17.

Museum of Southwestern Biology. Occurrence dataset

https://doi.org/10.15468/oirgxw accessed via GBIF.org on 2018-08-29..

Accessed from R via rgbif (https://github.com/ropensci/rgbif) on

2018-08-29

Rights:

[[16]]

<<rgbif citation>>

Citation: Royal Belgian Institute of Natural Sciences (2017). RBINS DaRWIN.

Occurrence dataset https://doi.org/10.15468/qxy4mc accessed via

GBIF.org on 2018-08-29.. Accessed from R via rgbif

(https://github.com/ropensci/rgbif) on 2018-08-29

Rights:

[[17]]

<<rgbif citation>>

Citation: Mateke C, Mulwanda S, Nachinga D (2018). Livingstone Museum Bat

Collection. Livingstone Museum. Occurrence dataset

https://doi.org/10.15468/i6cor7 accessed via GBIF.org on 2018-08-29..

Accessed from R via rgbif (https://github.com/ropensci/rgbif) on

2018-08-29

Rights:

**8.) *Rousettus aegyptiacus***

[[1]]

<<rgbif citation>>

Citation: iNaturalist.org (2018). iNaturalist Research-grade Observations.

Occurrence dataset https://doi.org/10.15468/ab3s5x accessed via

GBIF.org on 2018-08-29.. Accessed from R via rgbif

(https://github.com/ropensci/rgbif) on 2018-08-29

Rights:

[[2]]

<<rgbif citation>>

Citation: Musila S, Syingi R, Zuhura A, Gichuki N (2018). Distribution and

abundance of different bat species in the interior of Arabuko-Sokoke

Forest and adjacent farmlands in Gede, Kilifi County, Kenya. Version

1.2. National Museums of Kenya. Occurrence dataset

https://doi.org/10.15468/zekkpx accessed via GBIF.org on 2018-08-29..

Accessed from R via rgbif (https://github.com/ropensci/rgbif) on

2018-08-29

Rights:

[[3]]

<<rgbif citation>>

Citation: Grant S, Ferguson A (2018). Field Museum of Natural History

(Zoology) Mammal Collection. Version 9.5. Field Museum. Occurrence

dataset https://doi.org/10.15468/n4zgxw accessed via GBIF.org on

2018-08-29.. Accessed from R via rgbif

(https://github.com/ropensci/rgbif) on 2018-08-29

Rights:

[[4]]

<<rgbif citation>>

Citation: Olson L (2018). UAM Mammal Collection (Arctos). Version 34.16.

University of Alaska Museum of the North. Occurrence dataset

https://doi.org/10.15468/lbixob accessed via GBIF.org on 2018-08-29..

Accessed from R via rgbif (https://github.com/ropensci/rgbif) on

2018-08-29

Rights:

[[5]]

<<rgbif citation>>

Citation: naturgucker.de. naturgucker. Occurrence dataset

https://doi.org/10.15468/uc1apo accessed via GBIF.org on 2018-08-29..

Accessed from R via rgbif (https://github.com/ropensci/rgbif) on

2018-08-29

Rights:

[[6]]

<<rgbif citation>>

Citation: Esselstyn J (2017). LSUMZ Mammals Collection. Louisiana State

University Museum of Natural Science. Occurrence dataset

https://doi.org/10.15468/wxiqf6 accessed via GBIF.org on 2018-08-29..

Accessed from R via rgbif (https://github.com/ropensci/rgbif) on

2018-08-29

Rights:

[[7]]

<<rgbif citation>>

Citation: Mateke C, Mulwanda S, Nachinga D (2018). Livingstone Museum Bat

Collection. Livingstone Museum. Occurrence dataset

https://doi.org/10.15468/i6cor7 accessed via GBIF.org on 2018-08-29..

Accessed from R via rgbif (https://github.com/ropensci/rgbif) on

2018-08-29

Rights:

[[8]]

<<rgbif citation>>

Citation: KINGBO A, KIKI P P B (2016). Census of the animals of Benin.

Version 1.2. GBIF Benin. Occurrence dataset

https://doi.org/10.15468/h7rqo9 accessed via GBIF.org on 2018-08-29..

Accessed from R via rgbif (https://github.com/ropensci/rgbif) on

2018-08-29

Rights: Public Domain (CC0 1.0)

[[9]]

<<rgbif citation>>

Citation: Yêhouénou Tessi D R (2017). List of animalia, fungi and plant

species recorded through naturalist observations and research

activities in Benin. Data mobilized in the framework of a JRS

Biodiversity Foundation project of Benin. Laboratory of Forest Sciences

(University of Abomey-Calavi). Occurrence dataset

https://doi.org/10.15468/22kwre accessed via GBIF.org on 2018-08-29..

Accessed from R via rgbif (https://github.com/ropensci/rgbif) on

2018-08-29

Rights:

[[10]]

<<rgbif citation>>

Citation: Orrell T, Hollowell T (2018). NMNH Extant Specimen Records.

Version 1.19. National Museum of Natural History, Smithsonian

Institution. Occurrence dataset https://doi.org/10.15468/hnhrg3

accessed via GBIF.org on 2018-08-29.. Accessed from R via rgbif

(https://github.com/ropensci/rgbif) on 2018-08-29

Rights:

[[11]]

<<rgbif citation>>

Citation: MNHN - Museum national d'Histoire naturelle (2018). The mammals

collection (ZM) of the Muséum national d'Histoire naturelle (MNHN -

Paris). Version 43.77. Occurrence dataset

https://doi.org/10.15468/j0xw9i accessed via GBIF.org on 2018-08-29..

Accessed from R via rgbif (https://github.com/ropensci/rgbif) on

2018-08-29

Rights:

[[12]]

<<rgbif citation>>

Citation: Kiki P, Ganglo J (2017). Census of the threatened species of

Benin.. Version 1.5. GBIF Benin. Occurrence dataset

https://doi.org/10.15468/fbbbfl accessed via GBIF.org on 2018-08-29..

Accessed from R via rgbif (https://github.com/ropensci/rgbif) on

2018-08-29

Rights:

[[13]]

<<rgbif citation>>

Citation: European Molecular Biology Laboratory (EMBL) (2014).

Geographically tagged INSDC sequences. Occurrence dataset

https://doi.org/10.15468/cndomv accessed via GBIF.org on 2018-08-29..

Accessed from R via rgbif (https://github.com/ropensci/rgbif) on

2018-08-29

Rights:

[[14]]

<<rgbif citation>>

Citation: Conservation International. Rapid Assessment Program (RAP)

Biodiversity Survey Database. Occurrence dataset

https://doi.org/10.15468/tsrjm0 accessed via GBIF.org on 2018-08-29..

Accessed from R via rgbif (https://github.com/ropensci/rgbif) on

2018-08-29

Rights:

[[15]]

<<rgbif citation>>

Citation: Israel Nature and Parks Authority. Israel Nature and Parks

Authority. Occurrence dataset https://doi.org/10.15468/cmnjt1 accessed

via GBIF.org on 2018-08-29.. Accessed from R via rgbif

(https://github.com/ropensci/rgbif) on 2018-08-29

Rights:

[[16]]

<<rgbif citation>>

Citation: Horvitz N, Tsoar A (2015). BioGIS - Bats - Asaf Tsoar database.

Israel Nature and Parks Authority. Occurrence dataset

https://doi.org/10.15468/jmluox accessed via GBIF.org on 2018-08-29..

Accessed from R via rgbif (https://github.com/ropensci/rgbif) on

2018-08-29

Rights: Copyright 2014 BioGIS. Free for use by all individuals provided that

the owners are acknowledged in any use or publication.

[[17]]

<<rgbif citation>>

Citation: Flannery M, Fong J (2018). CAS Mammalogy (MAM). Version 133.136.

California Academy of Sciences. Occurrence dataset

https://doi.org/10.15468/dhbozg accessed via GBIF.org on 2018-08-29..

Accessed from R via rgbif (https://github.com/ropensci/rgbif) on

2018-08-29

Rights:

[[18]]

<<rgbif citation>>

Citation: Horvitz N (2015). BioGIS - Bats - SPNI. Israel Nature and Parks

Authority. Occurrence dataset https://doi.org/10.15468/1jzp2d accessed

via GBIF.org on 2018-08-29.. Accessed from R via rgbif

(https://github.com/ropensci/rgbif) on 2018-08-29

Rights: Copyright 2014 BioGIS. Free for use by all individuals provided that

the owners are acknowledged in any use or publication.

[[19]]

<<rgbif citation>>

Citation: Slade N (2018). KUBI Mammalogy Collection. Version 26.13.

University of Kansas Biodiversity Institute. Occurrence dataset

https://doi.org/10.15468/a3woj7 accessed via GBIF.org on 2018-08-29..

Accessed from R via rgbif (https://github.com/ropensci/rgbif) on

2018-08-29

Rights:

[[20]]

<<rgbif citation>>

Citation: Tushabe H (2018). National Biodiversity Data Bank. Observation

records, 1900-2014. Version 1.2. National Biodiversity Data Bank.

Occurrence dataset https://doi.org/10.15468/djzgie accessed via

GBIF.org on 2018-08-29.. Accessed from R via rgbif

(https://github.com/ropensci/rgbif) on 2018-08-29

Rights:

[[21]]

<<rgbif citation>>

Citation: Bradley J (2018). UWBM Mammalogy Collection (Arctos). University

of Washington Burke Museum. Occurrence dataset

https://doi.org/10.15468/qziy3w accessed via GBIF.org on 2018-08-29..

Accessed from R via rgbif (https://github.com/ropensci/rgbif) on

2018-08-29

Rights:

[[22]]

<<rgbif citation>>

Citation: Biological Station of Doñana (CSIC) (2018). Estacion Biologica

Donana - CSIC, Mammal Collection. Occurrence dataset

https://doi.org/10.15468/fwrsxr accessed via GBIF.org on 2018-08-29..

Accessed from R via rgbif (https://github.com/ropensci/rgbif) on

2018-08-29

Rights:

[[23]]

<<rgbif citation>>

Citation: Staatliche Naturwissenschaftliche Sammlungen Bayerns. The Mammalia

Collection at the Staatssammlung für Anthropologie und Paläoanatomie

München. Occurrence dataset https://doi.org/10.15468/elc2yx accessed

via GBIF.org on 2018-08-29.. Accessed from R via rgbif

(https://github.com/ropensci/rgbif) on 2018-08-29

Rights:

[[24]]

<<rgbif citation>>

Citation: Garner H (2016). TTU Mammals Collection. Version 9.1. Museum of

Texas Tech University (TTU). Occurrence dataset

https://doi.org/10.15468/yocqyp accessed via GBIF.org on 2018-08-29..

Accessed from R via rgbif (https://github.com/ropensci/rgbif) on

2018-08-29

Rights:

[[25]]

<<rgbif citation>>

Citation: Thompson C (2016). UMMZ Mammal Collection. Version 8.1. University

of Michigan Museum of Zoology. Occurrence dataset

https://doi.org/10.15468/dx3rcj accessed via GBIF.org on 2018-08-29..

Accessed from R via rgbif (https://github.com/ropensci/rgbif) on

2018-08-29

Rights:

[[26]]

<<rgbif citation>>

Citation: Millen B, Lim B (2018). Mammalogy Collection - Royal Ontario

Museum. Version 11.5. Royal Ontario Museum. Occurrence dataset

https://doi.org/10.15468/2rlrvh accessed via GBIF.org on 2018-08-29..

Accessed from R via rgbif (https://github.com/ropensci/rgbif) on

2018-08-29

Rights:

[[27]]

<<rgbif citation>>

Citation: Feeney R (2018). LACM Vertebrate Collection. Version 18.2. Natural

History Museum of Los Angeles County. Occurrence dataset

https://doi.org/10.15468/77rmwd accessed via GBIF.org on 2018-08-29..

Accessed from R via rgbif (https://github.com/ropensci/rgbif) on

2018-08-29

Rights:

[[28]]

<<rgbif citation>>

Citation: Cook J (2018). MSB Mammal Collection (Arctos). Version 35.17.

Museum of Southwestern Biology. Occurrence dataset

https://doi.org/10.15468/oirgxw accessed via GBIF.org on 2018-08-29..

Accessed from R via rgbif (https://github.com/ropensci/rgbif) on

2018-08-29

Rights:

[[29]]

<<rgbif citation>>

Citation: Muséum d'histoire naturelle de la Ville de Genève - MHNG. Mammals

housed at MHNG, Geneva. Occurrence dataset

https://doi.org/10.15468/lwu4fj accessed via GBIF.org on 2018-08-29..

Accessed from R via rgbif (https://github.com/ropensci/rgbif) on

2018-08-29

Rights: The use of the data is allowed only for non-profit scientific use

and for non-profit nature conservation purpose.

[[30]]

<<rgbif citation>>

Citation: Royal Belgian Institute of Natural Sciences (2017). RBINS DaRWIN.

Occurrence dataset https://doi.org/10.15468/qxy4mc accessed via

GBIF.org on 2018-08-29.. Accessed from R via rgbif

(https://github.com/ropensci/rgbif) on 2018-08-29

Rights:

[[31]]

<<rgbif citation>>

Citation: Trombone T (2016). AMNH Mammal Collections. American Museum of

Natural History. Occurrence dataset https://doi.org/10.15468/wu3poe

accessed via GBIF.org on 2018-08-29.. Accessed from R via rgbif

(https://github.com/ropensci/rgbif) on 2018-08-29

Rights:

[[32]]

<<rgbif citation>>

Citation: Australian Museum (2017). Australian Museum provider for OZCAM.

Occurrence dataset https://doi.org/10.15468/e7susi accessed via

GBIF.org on 2018-08-29.. Accessed from R via rgbif

(https://github.com/ropensci/rgbif) on 2018-08-29

Rights:

[[33]]

<<rgbif citation>>

Citation: Gall L (2018). Vertebrate Zoology Division - Mammalogy, Yale

Peabody Museum. Yale University Peabody Museum. Occurrence dataset

https://doi.org/10.15468/4mm6uc accessed via GBIF.org on 2018-08-29..

Accessed from R via rgbif (https://github.com/ropensci/rgbif) on

2018-08-29

Rights:

[[34]]

<<rgbif citation>>

Citation: Bucci M (2016). UAZ Mammals. Version 5.1. University of Arizona

Museum of Natural History. Occurrence dataset

https://doi.org/10.15468/2swesj accessed via GBIF.org on 2018-08-29..

Accessed from R via rgbif (https://github.com/ropensci/rgbif) on

2018-08-29

Rights:

[[35]]

<<rgbif citation>>

Citation: Shugart G (2016). PSM Vertebrates Collection. Version 8.1. James

R. Slater Museum of Natural History. Occurrence dataset

https://doi.org/10.15468/3oaz5o accessed via GBIF.org on 2018-08-29..

Accessed from R via rgbif (https://github.com/ropensci/rgbif) on

2018-08-29

Rights:

[[36]]

<<rgbif citation>>

Citation: Queensland Museum (2018). Queensland Museum provider for OZCAM.

Occurrence dataset https://doi.org/10.15468/lotsye accessed via

GBIF.org on 2018-08-29.. Accessed from R via rgbif

(https://github.com/ropensci/rgbif) on 2018-08-29

Rights:

[[37]]

<<rgbif citation>>

Citation: Braun J, King P (2018). Mammals Specimens. Sam Noble Oklahoma

Museum of Natural History. Occurrence dataset

https://doi.org/10.15468/fmuion accessed via GBIF.org on 2018-08-29..

Accessed from R via rgbif (https://github.com/ropensci/rgbif) on

2018-08-29

Rights:

[[38]]

<<rgbif citation>>

Citation: Conroy C (2018). MVZ Mammal Collection (Arctos). Version 35.17.

Museum of Vertebrate Zoology. Occurrence dataset

https://doi.org/10.15468/uwudf9 accessed via GBIF.org on 2018-08-29..

Accessed from R via rgbif (https://github.com/ropensci/rgbif) on

2018-08-29

Rights:

[[39]]

<<rgbif citation>>

Citation: Museum and Art Gallery of the Northern Territory (2017). Northern

Territory Museum and Art Gallery provider for OZCAM. Occurrence dataset

https://doi.org/10.15468/giro3a accessed via GBIF.org on 2018-08-29..

Accessed from R via rgbif (https://github.com/ropensci/rgbif) on

2018-08-29

Rights:

[[40]]

<<rgbif citation>>

Citation: Western Australian Museum (2017). Western Australian Museum

provider for OZCAM. Occurrence dataset https://doi.org/10.15468/5qt0dm

accessed via GBIF.org on 2018-08-29.. Accessed from R via rgbif

(https://github.com/ropensci/rgbif) on 2018-08-29

Rights:

[[41]]

<<rgbif citation>>

Citation: South Australian Museum (2018). South Australian Museum Australia

provider for OZCAM. Occurrence dataset https://doi.org/10.15468/wz4rrh

accessed via GBIF.org on 2018-08-29.. Accessed from R via rgbif

(https://github.com/ropensci/rgbif) on 2018-08-29

Rights:

[[42]]

<<rgbif citation>>

Citation: Natural History Museum (2018). Natural History Museum (London)

Collection Specimens. Occurrence dataset

https://doi.org/10.5519/0002965 accessed via GBIF.org on 2018-08-29..

Accessed from R via rgbif (https://github.com/ropensci/rgbif) on

2018-08-29

Rights:

[[43]]

<<rgbif citation>>

Citation: Turpel A, Walisch T (2018). Collections and observation data

National Museum of Natural History Luxembourg. Musée national

d'histoire naturelle Luxembourg. Occurrence dataset

https://doi.org/10.15468/s2iu7d accessed via GBIF.org on 2018-08-29..

Accessed from R via rgbif (https://github.com/ropensci/rgbif) on

2018-08-29

Rights:

[[44]]

<<rgbif citation>>

Citation: Marine Science Institute, UCSB. Paleobiology Database. Occurrence

dataset https://doi.org/10.15468/2durgn accessed via GBIF.org on

2018-08-29.. Accessed from R via rgbif

(https://github.com/ropensci/rgbif) on 2018-08-29

Rights:

[[45]]

<<rgbif citation>>

Citation: Nelson A, Johnson N (2018). Ohio State University Tetrapod

Division - Mammal Collection (OSUM). Version 93.36. Museum of

Biological Diversity, The Ohio State University. Occurrence dataset

https://doi.org/10.15468/jyhwea accessed via GBIF.org on 2018-08-29..

Accessed from R via rgbif (https://github.com/ropensci/rgbif) on

2018-08-29

Rights:

**Supplementary material S3: Occurrences of bat species taken from African Chiroptera Report**^2,3^

a) *Epomops franqueti*  b) *Hypsignathus monstrosus* c) *Myonycteris torquata* d) *Eidolon helvum*

e) *Epomophorus gambianus*  f) *Lissonycteris angolensis* g) *Micropteropus pusillus* h) *Mops condylurus* i) *Rousettus aegyptiacus*

**Supplementary material S4: Variable Importance and AUC values of the models for the nine bat species.** We used a maximum entropy approach conducted in the freeware MaxEnt^5^ to model the habitat suitability for the nine bat species.

| Species | AUC | Variable | Contribution | Variable | Contribution | Variable | Contribution | Variable | Contribution | Variable | Contribution | Variable | Contribution | Variable | Contribution | Variable | Contribution | Variable | Contribution |
| --- | --- | --- | --- | --- | --- | --- | --- | --- | --- | --- | --- | --- | --- | --- | --- | --- | --- | --- | --- |
| *Epomops franqueti* | 0,9352 | landcover | 40,44 | bio04 | 30,88 | bio15 | 11,4 | bio06 | 5,83 | bio05 | 5,13 | bio14 | 2,94 | bio12 | 2,14 | bio01 | 0,69 | bio13 | 0,54 |
| *Hypsignathus monstrosus* | 0,9381 | bio04 | 35,88 | landcover | 27,51 | bio12 | 9,45 | bio14 | 8,88 | bio06 | 6,50 | bio15 | 6,05 | bio05 | 3,73 | bio01 | 1,69 | bio13 | 0,32 |
| *Myonycteris torquata* | 0,9338 | landcover | 50,6 | bio04 | 18,31 | bio14 | 8,97 | bio13 | 7,16 | bio15 | 5,68 | bio05 | 4,40 | bio12 | 2,86 | bio01 | 1,95 | bio06 | 0,07 |
| *Eidolon helvum* | 0,8481 | landcover | 62,43 | bio04 | 19,99 | bio05 | 4,68 | bio12 | 3,18 | bio14 | 2,43 | bio06 | 2,20 | bio01 | 1,99 | bio13 | 1,61 | bio15 | 1,49 |
| *Epomophorus gambianus* | 0,891 | landcover | 60,4 | bio06 | 13,04 | bio04 | 8,14 | bio05 | 5,63 | bio01 | 3,94 | bio12 | 3,82 | bio14 | 2,69 | bio13 | 1,85 | bio15 | 0,50 |
| *Lissonycteris angolensis* | 0,907 | bio04 | 60,8 | landcover | 9,1 | bio15 | 7,9 | bio05 | 7,8 | bio12 | 7,2 | bio14 | 5,3 | bio01 | 1 | bio06 | 0,9 | bio13 | 0,1 |
| *Micropteropus pusillus* | 0,8956 | landcover | 48,39 | bio04 | 26,25 | bio06 | 12,15 | bio12 | 4,11 | bio05 | 2,92 | bio15 | 2,90 | bio14 | 2,22 | bio13 | 0,55 | bio01 | 0,52 |
| *Mops condylurus* | 0,8439 | landcover | 62,06 | bio04 | 26,91 | bio14 | 5,62 | bio15 | 2,79 | bio12 | 1,75 | bio06 | 0,40 | bio13 | 0,27 | bio05 | 0,19 | bio01 | 0,00 |
| *Rousettus aegyptiacus* | 0,8614 | landcover | 44,94 | bio05 | 15,96 | bio04 | 7,95 | bio14 | 7,64 | bio13 | 7,25 | bio06 | 7,05 | bio15 | 5,44 | bio01 | 2,90 | bio12 | 0,88 |

**Supplementary material S5: One variable response curves for the categorical land cover variable.** The modelling part was done with the freeware MaxEnt^5^ and the statistical programme R^6^.


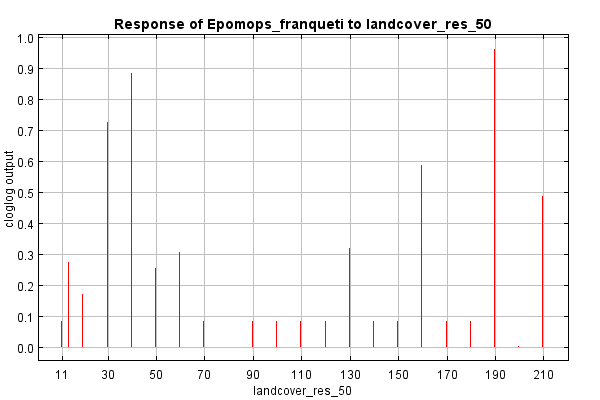

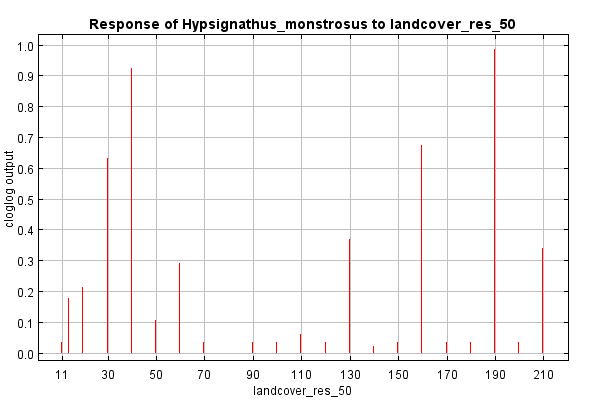

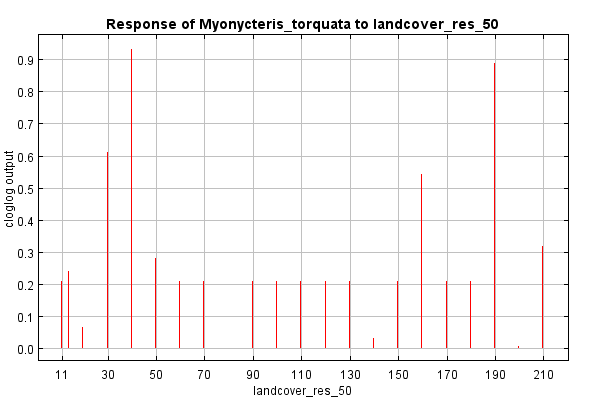

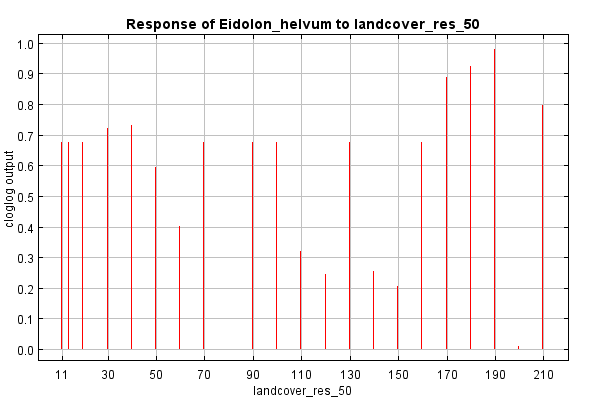


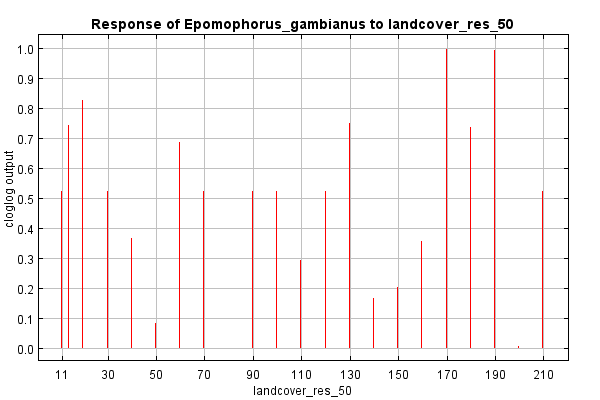

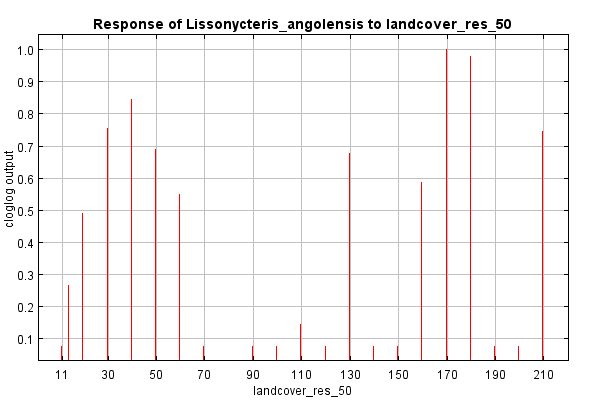

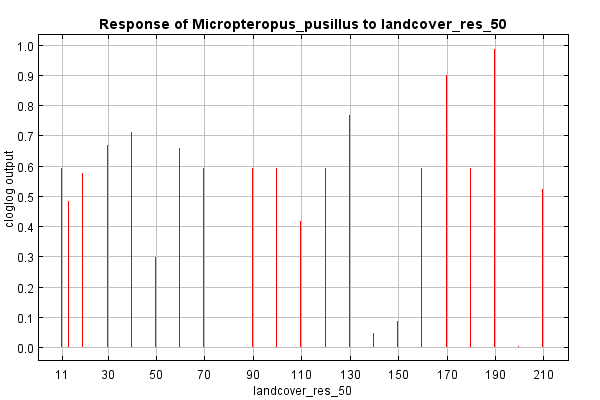

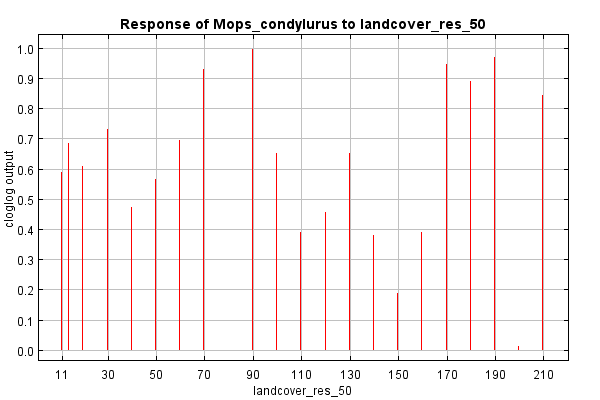

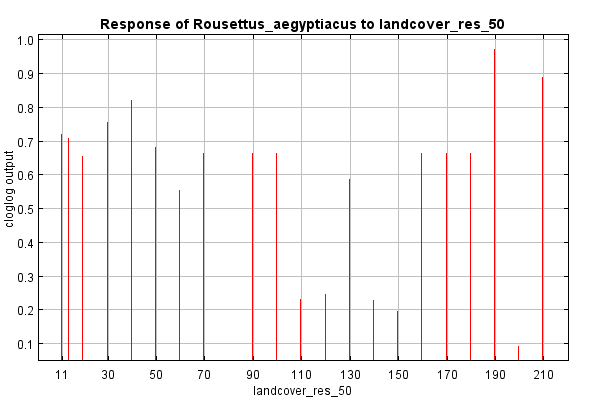


One variable response curves for the categorical land cover variable: a) *Epomops franqueti*, b) *Hypsignathus monstrosus*, c) *Myonycteris torquata*, d) *Eidolon helvum*, e) *Epomophorus gambianus*, f) *Lissonycteris angolensis*, g) *Micropteropus pusillus*, h)  *Mops condylurus*, i) *Rousettus aegyptiacus Mops condylurus.* Land cover classes: 11 – Post-flooding or irrigated croplands, 14 – Rainfed croplands, 20 – Mosaic Cropland (50-70%) / Vegetation (grassland, shrubland, forest) (20-50%), 30 – Mosaic Vegetation (grassland, shrubland, forest) (50-70%) / Cropland (20-50%), 40 – Closed to open (>15%) broadleaved evergreen and/or semi-deciduous forest (>5m), 50 – Closed (>40%) broadleaved deciduous forest (>5m), 60 – Open (15-40%) broadleaved deciduous forest (>5m), 70 – Closed (>40%) needleleaved evergreen forest (>5m), 90 – Open (15-40%) needleleaved deciduous or evergreen forest (>5m), 100 – Closed to open (>15%) mixed broadleaved and needleleaved forest (>5m), 110 – Mosaic Forest/Shrubland (50-70%) / Grassland (20-50%), 120 – Mosaic Grassland (50-70%) / Forest/Shrubland (20-50%), 130 – Closed to open (>15%) shrubland (<5m), 140 – Closed to open (>15%) grassland, 150 – Sparse (<15%) vegetation (woody vegetation, shrubs, grassland), 160 – Closed (>40%) broadleaved forest regularly flooded – Fresh water, 170 – Closed (>40%) broadleaved semi-deciduous and/or evergreen forest regularly flooded – Saline water, 180 – Closed to open (>15%) vegetation (grassland, shrubland, woody vegetation) on regularly flooded or waterlogged soil – Fresh, brackish or saline water, 190 – Artificial surfaces and associated areas (urban areas >50%), 200 – Bare areas, 210 – Water bodies, 220 – Permanent snow and ice, 230 – No data.

**Supplementary material S6: References of the exact IUCN polygons (as of January 2020)**^7^.

- ***Epomops franqueti*:**

Kityo, R. & Nalikka, B. 2016. *Epomops franqueti* . *The IUCN Red List of Threatened Species* 2016: e.T7909A22116503 <https://dx.doi.org/10.2305/IUCN.UK.2016-1.RLTS.T7909A22116503.en>.

- ***Hypsignathus monstrosus:***

Tanshi, I. 2016. *Hypsignathus monstrosus* (errata version published in 2017). *The IUCN Red List of Threatened Species* 2016: e.T10734A115098825. <https://dx.doi.org/10.2305/IUCN.UK.2016-3.RLTS.T10734A21999919.en>.

- ***Myonycteris torquata:***

Bakwo Fils, E.M. & Kaleme, P. 2016. *Myonycteris torquata* . *The IUCN Red List of Threatened Species* 2016: e.T84463104A22046504. <https://dx.doi.org/10.2305/IUCN.UK.2016-1.RLTS.T84463104A22046504.en>.

- ***Eidolon helvum:***

Mickleburgh, S., Hutson, A.M., Bergmans, W., Fahr, J. & Racey, P.A. 2008. *Eidolon helvum*. *The IUCN Red List of Threatened Species* 2008: e.T7084A12824968. <https://dx.doi.org/10.2305/IUCN.UK.2008.RLTS.T7084A12824968.en>.

- ***Epomophorus gambianus:***

Tanshi, I. & Fahr, J. 2016. Epomophorus gambianus . The IUCN Red List of Threatened Species 2016: e.T7903A22122670. <https://dx.doi.org/10.2305/IUCN.UK.2016-2.RLTS.T7903A22122670.en>.

- ***Lissonycteris angolensis:***

Bergmans, W., Hutson, A.M., Mickleburgh, S. & Monadjem, A. 2017. *Lissonycteris angolensis* . *The IUCN Red List of Threatened Species* 2017: e.T44698A22073874. <https://dx.doi.org/10.2305/IUCN.UK.2017-2.RLTS.T44698A22073874.en>.

- ***Micropteropus pusillus:***

Bakwo Fils, E.M. & Kaleme, P. 2016. *Micropteropus pusillus* . *The IUCN Red List of Threatened Species* 2016: e.T13402A22126384. <https://dx.doi.org/10.2305/IUCN.UK.2016-1.RLTS.T13402A22126384.en>.

- ***Mops condylurus:***

Monadjem, A., Cotterill, F., Hutson, A.M., Mickleburgh, S. & Bergmans, W. 2017. *Mops condylurus* . *The IUCN Red List of*

*Threatened Species* 2017: e.T13838A22075340. <https://dx.doi.org/10.2305/IUCN.UK.2017-2.RLTS.T13838A22075340.en>.

- ***Rousettus aegyptiacus:***

Korine, C. 2016. *Rousettus aegyptiacus* . *The IUCN Red List of Threatened Species* 2016: e.T29730A22043105. <https://dx.doi.org/10.2305/IUCN.UK.2016-2.RLTS.T29730A22043105.en>.

**References supplementary material:**

1. GBIF. Global Biodiversity Information Facility. GBIF Home Page. (2018).

2. ACR. *African Chiroptera Report 2018. AfricanBats NPC*. (2018). doi:10.13140/RG.2.2.18794.82881

3. ACR. *African Chiroptera Report 2019. AfricanBats NPC*. (2019). doi:10.13140/RG.2.2.27442.76482.1990-6471

4. ESRI. Environmental Systems Research Institute (ESRI). ArcGIS Release 10.6. Redlands, CA (https://www.esri.com/en-us/home). (2018).

5. Phillips, S. J., Dudík, M. & Schapire, R. E. [Internet] Maxent software for modeling species niches and distributions (Version 3.4.1). Available from url: http://biodiversityinformatics.amnh.org/open_source/maxent/. Accessed in 2019. (2017).

6. R Core Team. R: A language and environment for statistical computing. R Foundation for Statistical Computing, Vienna, Austria. URL https://www.R-project.org/. (2017).

7. IUCN. IUCN (International Union for Conservation of Nature and Natural Resources). The IUCN Red List of Threatened Species. Version 2019-3. <https://www.iucnredlist.org>. (2020). Available at: https://www.iucnredlist.org/search.
